# Mammary Leukocyte‐Assisted Nanoparticle Transport Enhances Targeted Milk Trace Mineral Delivery

**DOI:** 10.1002/advs.202200841

**Published:** 2022-06-30

**Authors:** Jie Cai, Jie Peng, Xinwei Zang, Juan Feng, Ruocheng Li, Peng Ren, Bingzhu Zheng, Jiaying Wang, Juan Wang, Mi Yan, Jianxin Liu, Renren Deng, Diming Wang

**Affiliations:** ^1^ Institute of Dairy Science College of Animal Sciences MOE Key Laboratory of Molecular Animal Nutrition Zhejiang University Hangzhou 310029 P. R. China; ^2^ School of Materials Science and Engineering State Key Laboratory of Silicon Materials Institute for Composites Science Innovation Zhejiang University Hangzhou 310027 P. R. China; ^3^ Institute of Environmental Health MOE Key Laboratory of Environmental Remediation and Ecosystem Health College of Environmental & Resource Sciences Zhejiang University Hangzhou 310058 P. R. China; ^4^ Department of Medical Oncology The First Affiliated Hospital School of Medicine Zhejiang University Hangzhou 310003 P. R. China

**Keywords:** leukocyte, mammary gland, milk, mineral nanoparticles, nutrient delivery

## Abstract

Nanoparticles are applied as versatile platforms for drug/gene delivery in many applications owing to their long‐retention and specific targeting properties in living bodies. However, the delivery mechanism and the beneficial effect of nanoparticle‐retention in many organisms remain largely uncertain. Here, the transport and metabolism of mineral nanoparticles in mammary gland during lactation are explored. It is shown that maternal intravenous administration of iron oxide nanoparticles (IONPs; diameter: ≈11.0 nm, surface charge: −29.1 mV, surface area: 1.05 m^2^ g^−1^) provides elevated iron delivery to mammary gland and increased iron secretion into breast milk, which is inaccessible by classical iron‐ion transport approaches such as the transferrin receptor‐mediated endocytic pathway. Mammary macrophages and neutrophils are found to play dominant roles in uptake and delivery of IONPs through an unconventional leukocyte‐assisted iron secretion pathway. This pathway bypasses the tight iron concentration regulation of liver hepcidin‐ferroportin axis and mammary epithelial cells to increase milk iron‐ion content derived from IONPs. This work provides keen insight into the metabolic pathway of nanoparticles in mammary gland while offering a new scheme of nutrient delivery for neonate metabolism regulation by using nanosized nutrients.

## Graphical abstract

Iron oxide nanoparticles (IONPs) are captured by mammary‐resident leukocytes, exhibiting more iron influx into lactating mammary glands than iron ion. IONPs are metabolized by mainly mammary leukocytes into iron ions, which are still kept within these cells. These cells migrate into milk and meanwhile exert effective iron transfer in a safe intracellular iron form, displaying a novel leukocyte‐assisted nutrient delivery.

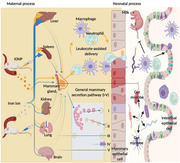

## 1 Introduction

Milk is the universal preferred nutrition for newborn infants due to its ability to provide complete nutrition and bioactive factors.^[^ ^1^ ^]^ As the sole nutritional source for newborns, it is extremely important to fortify specific milk components when they are scarce to promote healthy growth of neonates. Because the way that milk is fortified through maternal manipulation depends largely on the concentration of milk substrates delivered to the mammary glands,^[^ ^2^, ^3^ ^]^ the supply of most nutritional components in breast milk such as lipid, protein, vitamin, and other micronutrients (e.g., nicotinamide) can be enhanced by increasing maternal nutrient supplements either orally or intravenously.^[^ ^4^, ^5^, ^6^, ^7^, ^8^ ^]^ However, essentially improved trace mineral elements (such as zinc, iron, and copper) in milk via maternal addition is not wieldy,^[^ ^9^, ^10^, ^11^, ^12^, ^13^ ^]^ due to the stringent concentration control of these elements in maternal circulation.

For most of the trace mineral elements, liver and homeostasis axis orchestrate their systemic balances by hepatic portal vein filtration and negative feedback to maintain normal physiology. For instance, supplied iron is mostly sensed and sequestered in liver by coordinating with the production of iron‐regulating hepcidin‐ferroportin (FPN) axis. It regulates the concentration of circulating iron in a narrow range.^[^ ^10^, ^13^, ^14^, ^15^, ^16^ ^]^ Meanwhile, the secretion of iron, zinc, and copper into milk is tightly regulated and maintained by mammary epithelial cells (MECs) during lactation.^[^ ^10^, ^15^ ^]^ These regulations cause the concentration of trace mineral elements in breast milk are 1) remarkably stable at each of the different stages of lactation and decreasing as lactation progresses; 2) independent of maternal mineral intake or maternal mineral manipulations.^[^ ^4^, ^16^ ^]^ Thus, manipulating the transport and secretion of trace mineral elements in mammary gland and milk remains a daunting challenge.

The stringent concentration regulation of trace mineral elements can be potentially addressed by using mineral nanoparticles as nutrient supplies. Compared with conventional ionic mineral sources, synthetic mineral nanoparticles have distinctly different metabolic pathways.^[^ ^17^, ^18^ ^]^ Nanoparticles are easily captured by organs with enriched reticuloendothelial systems, therefore they may naturally accumulate in mammary gland during circulation.^[^ ^19^, ^20^ ^]^ Nanoparticles can also undergo specific cellular transport paths which bypass conventional transport receptors via various cellular vehicles such as immune cells, immunocytes' cell membranes, leukocyte‐derived extracellular vesicles, or exosomes.^[^ ^21^, ^22^ ^]^ These properties offer opportunities to develop delivery strategies with improved distribution and targeting abilities.

**Figure 1:**
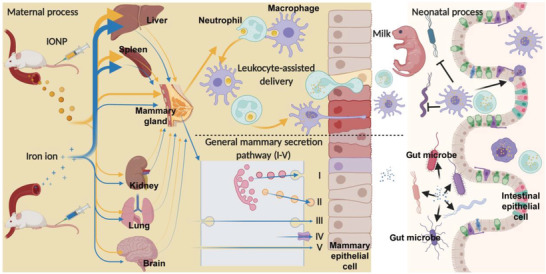
Illustration of a novel pathway of mammary nutrient delivery through maternal nanoparticle administration. The administrated IONPs are captured by mammary‐resident leukocytes including macrophages and neutrophils, exhibiting more iron influx into the lactating mammary glands than conditional iron supplements (iron ion, such as FeC) with the same iron‐treated level. The IONPs are in situ metabolized by mainly mammary leukocytes into iron ions, which are still kept within these cells. As these cells migrate into milk and meanwhile exert iron transfer, we call this pathway as leukocyte‐assisted delivery. This pathway has advantages over the general mammary secretion pathway, including an effective iron transfer and a safe intracellular iron form.

Here, we report a novel route of mammary nutrient delivery through maternal nanoparticle administration. We used iron oxide nanoparticles (IONPs), which successfully improved milk iron and became a potential window of maternal manipulation (**Figure** **1**). In the nanoparticle delivery pathway, mammary leukocytes including macrophages and neutrophils exhibit direct IONP capture in the lactating mammary gland and bypass barriers of liver hepcidin‐FPN negative feedback axis and MECs. These activated leukocytes bio‐transform captured IONPs into iron‐ion which largely increase the iron content of breast milk by a factor of 64%. When these iron‐containing milk cells were orally delivered to neonates, iron was segregated intracellularly by leukocytes until metabolized by terminal organs. Importantly, the iron nanoparticle‐to‐milk delivery pathway avoids adverse effects by direct iron feeding to neonate, such as obstipation, diarrhea, abdominal pain, skin irritation, nausea, vomiting, and allergic reactions,^[^ ^23^, ^24^ ^]^ and reduces infection risk induced by pathogens competing for iron. This method largely enhances neonate iron delivery, showing significant therapeutic effect for both anemia and bacterial infection without causing any observed adverse effects. Our findings provided a new pathway of maternal nutrient delivery effectively and safely for neonatal mineral supply.

## 2 Results and Discussion

### 2.1 Landscape of IONP Distribution in Main Organs of Lactating Mice

**Figure 2:**
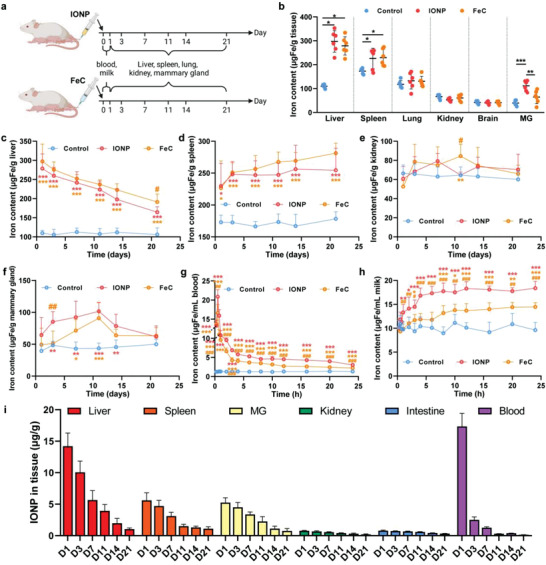
In vivo accumulation, degradation, and transfer of IONPs in lactating mice. a) Scheme of IONP and FeC administration (2.8 mg Fe kg^–1^ body weight) and sampling. b) Excess iron accumulated in organs 24 h after intravenous injection of IONPs, FeC, and PBS (as a control) was quantified by ICP‐MS. c–f) Quantity of total iron measured by ICP‐MS in c) liver, d) spleen, e) kidney, and f) mammary gland (MG) as a function of days after intravenous IONP or FeC injection. g,h) Entrance of the IONPs or FeC into g) blood and h) milk 24 h after administration was determined by ICP‐MS. i) Amount of IONPs in liver, spleen, mammary gland (MG), kidney, intestine, and blood as a function of days (D) after IONP administration was determined by ferromagnetic resonance spectra. Representative spectra can be seen in Figure S13 in the Supporting Information. The data are presented as the mean ± standard deviation (*n* = 6). b) *, **, and *** denote statistical significances, *p* < 0.05, *p* < 0.01, and *p* < 0.001, respectively. c–h) In comparison of IONP versus control (red color) or FeC versus control (orange color): *, **, and *** denote statistical significances, *p* < 0.05, *p* < 0.01, and *p* < 0.001, respectively; in comparison of IONP versus FeC (orange color): #, ##, and ### denote statistical significances, *p* < 0.05, *p* < 0.01, and *p* < 0.001, respectively.

**Figure 3:**
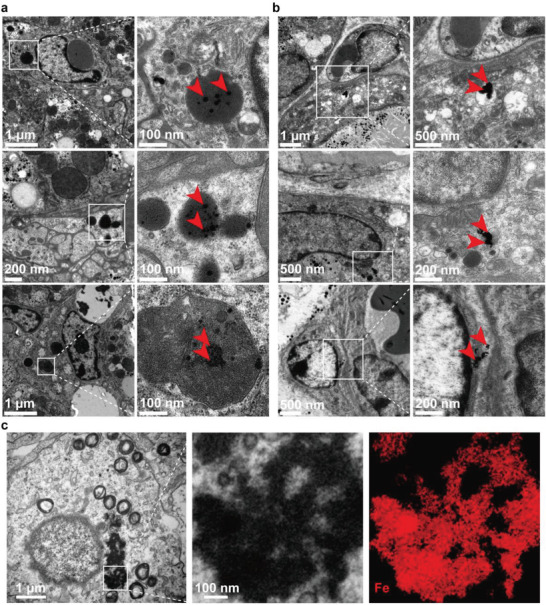
TEM pictures of liver and mammary gland in lactating mice intravenously injected with IONPs. a,b) TEM of a) liver and b) mammary gland in lactating mice after 1 (upper), 7 (middle), 11 (lower) days after intravenously injected with IONPs. Pictures in left are large‐scale views. Typical sites containing IONP uptake by cells are labeled in left and enlarged in right. The intracellular IONPs were annotated by red arrow. c) TEM pictures of mammary gland in lactating mice after 7 days after intravenously injected with IONPs (left). IONP‐laden sites in mammary epithelial cell are enlarged (middle). IONP‐laden sites are analyzed by EDX spectrum imaging, demonstrating that iron chemical map (right) colocalizes with the electron‐dense IONPs observed on the bright‐field image (middle).

We applied IONPs (Figure S1, Supporting Information) as the nanoparticulate iron source, and a clinical iron supplement, ferric carboxymaltose (FeC), as a control of a classical ionic iron source. The iron contents of main organs in lactating mice 24 h post‐injection of IONPs and FeC showed that both two iron supplements (2.8 mg Fe kg^–1^) significantly increased iron content of liver and spleen, which are centers of iron storage and metabolism (**Figure** **2**). However, only IONPs statistically increased iron content of mammary gland (*p* < 0.05). Time‐series iron fluctuation post‐injection showed that the total mass of iron accumulated in the liver by one‐dose of IONPs or FeC decreased with time, while the total mass of iron in the spleen increased, indicating a potential redistribution of iron after treatments and the final iron storage into spleen. The iron content was increased in the mammary gland with time until peak lactation (lactation day 11 in mice) and then slightly decreased to normal level when lactation was stopped. Short time‐series (within 24 h) iron fluctuation post‐injection showed that blood iron was rapidly increased after iron injection and then decreased over time, showing a first‐degree partition and uptake by cells or organs and demonstrating the iron removal from the blood. Meanwhile, milk iron content increased after both iron supplements, but more significant in IONP treatment than FeC. These results suggest that IONPs have a superior function in increasing iron content in milk. In addition, systematic iron homeostasis functions as barriers for the iron delivery to mammary gland and milk, indicated by the significantly increased iron in liver and spleen, and fast elimination of blood iron especially in the FeC treatment. More importantly, hepcidin‐FPN axis is more significantly influenced by FeC than IONPs in relative to the control (Figure S2, Supporting Information). In FeC treatment, plasma iron loading directly increased circulating transferrin‐bound iron (2Fe‐TF), which is sensed by hepatocytes and binds to transferrin receptors on the hepatocyte membrane,^[^ ^14^ ^]^ stimulating hepcidin expression via a functional interaction with the small mothers against decapentaplegic (SMAD) pathway. Hepcidin secreted by hepatocyte binds in turn to FPN, causing systematic FPN ubiquitination and degradation and sequesters the iron intracellularly. Thus, the iron is quickly removed from circulation and kept within organs with ability of iron storage via the systematic regulation of iron homeostasis during the FeC treatment, preventing the iron transport to mammary gland and milk.^[^ ^25^ ^]^ However, 2Fe‐TF cannot be formed directly from IONPs and fail to trigger the downstream pathway of SMAD signaling, resulting in an insufficient production of hepcidin and thus inactivated systematic iron regulation. Sequestered IONPs in liver release iron via biotransformation and may trigger intracellular regulation by transcriptional, posttranscriptional, posttranslational mechanism, such as RNF217‐related signaling in FPN downregulation, which were independent of hepcidin and did not trigger systematic iron sequestration.^[^ ^26^, ^27^, ^28^ ^]^ Moreover, the mammary gland acquires iron depending on mammary‐resident immune cells by capturing IONP directly, which is also independent of hepcidin‐FPN axis (**Figure** **3**). Thus, IONPs can bypass the recognition of hepcidin‐FPN axis and allows enhancing milk iron.

In addition to above results of total iron measured by inductively coupled plasma‐mass spectrometry (ICP‐MS), we further traced the IONPs in tissues by ferromagnetic resonance (FMR), enabling us to separate exogenous IONPs from endogenous iron pools and to gain better understandings of IONP biotransformation (Figure 2i). On day 1 post‐injection, we observed the accumulated nanoparticle gradient: liver (46.2%) > spleen (5.6%) > lactating mammary gland (1.4%) > intestine (1.1%) > kidney (0.3%). It is reasonable that developed reticuloendothelial systems in liver, spleen, intestine, and mammary‐resident immune cells contribute to the interception of IONPs in the blood (Figure 3). However, these processes are not occurred in the kidney which is lack of reticuloendothelial cells under normal physiology. Over the course of entire lactation period after IONP treatments, liver, spleen, kidney, and breast showed a significant decrease of their IONP content, reaching a low level (0.08–3.4%) when lactation was stopped.

Of note, a majority of IONPs are transformed in the liver. It is consistent, first, with the large uptake capacity of the liver compared to other organs and second, with the gradual disappearance of IONPs from the liver. Subsequently, the non‐IONP products of degradation are not stocked in the liver but mostly transferred to the spleen to increase the iron storage tank, probably in the non‐IONP form of ferritin or hemosiderin. Moreover, a part of iron was transported into mammary gland and excreted more into milk as the output, but less into kidney. These results demonstrated that milk iron was a preferential output for injected IONPs during lactation. Moreover, iron metabolic partitions are unlikely to be the only contributor to the increased milk iron by IONP, since administration of FeC at the same amount of iron increased milk iron much less than that of IONP, despite that the injected FeC did not even require a long period of biotransformation.

### 2.2 IONPs Function as Milk Iron Enhancers by Regulating Milk Cells

**Figure 4:**
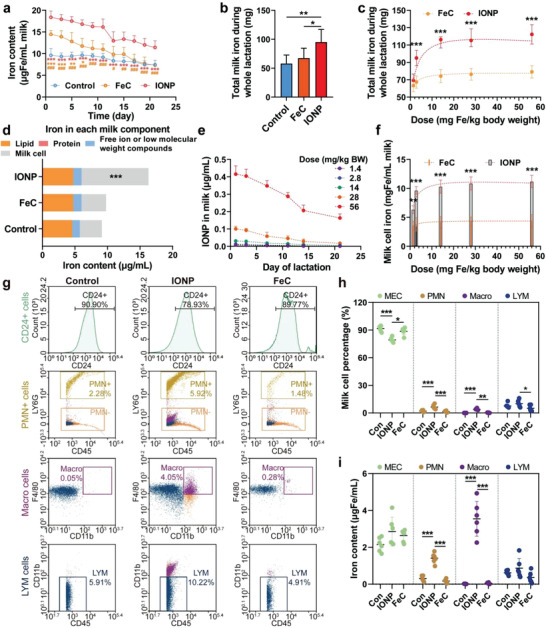
Enhancement effect of IONPs on milk iron. a) Milk iron content influenced by intravenous IONPs (2.8 mg Fe kg^–1^), FeC (2.8 mg Fe kg^–1^), or PBS (as a control) injection as a function of lactation days. b) Total milk iron during the whole lactation influenced by IONPs or FeC (2.8 mg Fe kg^–1^). c) Total milk iron during the whole lactation as a function of doses of IONPs or FeC. d) Iron in each milk component (lactation day 11–12) influenced by IONPs or FeC (2.8 mg Fe kg^–1^). e) Milk IONP content influenced by various doses of intravenous IONPs as a function of lactation days. f) Milk cell iron content (lactation day 11–12) as a function of doses of IONPs or FeC. g) Typical milk cell profiles (lactation day 11–12) influenced by intravenous IONPs or FeC injection (2.8 mg Fe kg^–1^). h) Cell percentages of different milk cell types after the treatments. i) Iron content in different milk cell types (lactation day 11–12) influenced by intravenous IONPs or FeC (lactation day 11–12). The iron content refers to total iron content for each cell type in a given volume of milk. MEC, mammary epithelial cell; PMN, neutrophil; Macro, macrophage; LYM, lymphocyte. The data are presented as the mean ± standard deviation (*n* = 6). *, **, and *** denote statistical significances, *p* < 0.05, *p* < 0.01, and *p* < 0.001, respectively.

We further quantified the enhancement effect of IONPs on milk iron. We noticed the gradually decreased milk iron content in both control (phosphate buffer saline, PBS) and iron supplement groups over time, whereas milk iron content was the highest in IONP‐treated group at each time point during the whole lactation (**Figure** **4**a). The FeC treatment only temporarily increased milk iron in the first 4 days, compared to the control group. Compared with the control group, entire milk iron during lactation was significantly enhanced by 64% through IONP treatment, but showed no significant changes through FeC treatment (Figure 4b). A dose‐dependent response of milk iron to maternal intravenous IONP treatment was further explored by extending the responsive dose (2.8 mg Fe kg^–1^) to 0.5–20 times (1.4–56 mg Fe kg^–1^) (Figure 4c). Compared with the limited response and effectiveness of milk iron regulation (63.4–79.1 mg Fe/lactation/mice) by FeC (1.4–56 mg Fe kg^–1^), milk iron showed a significant increase response (69.3–122.3 mg Fe/lactation/mice) to IONPs in a Lognormal dose‐dependent manner. Milk iron does not further increase when the injection dose of IONPs exceeds 14 mg Fe kg^–1^, indicating that the uptake and biotransformation of IONPs by the mammary gland has an upper bound.

The milk iron forms in the IONP treatment were further analyzed (Figure 4d). The majority of milk iron is non‐IONP form as IONPs in milk are only 0.0012–0.42 µg Fe mL^–1^ accounting for 0.0067–2.3% of total milk iron. These results indicated a biotransformation of IONPs by mammary glands. Under subcellular observation, IONPs in mammary cells were observed in nonspherical form, indicating an intracellular IONP degradation, whereas on same days IONPs in liver cells were mainly kept in an original sphere form (Figure 3). However, high doses of IONPs (over 14 mg Fe kg^–1^) still significantly increase the milk IONPs during the whole lactation, again showing an upper bound of dealing with IONPs by mammary glands (Figure 4e).

In general, the mammary iron was transported into milk by five pathways (I–V), corresponding to iron in milk proteins (I, III), iron in lipids (II), and free iron ion or iron in low molecular weight compounds (IV, V).^[^ ^3^ ^]^ Unexpectedly, iron secreted by these pathways had a minor contribution (3.7%) to the increased milk iron in IONP groups (2.8 mg Fe kg^–1^; Figure 4d). An exception to these pathways is the contribution of milk cell iron, which is generally ignored,^[^ ^29^ ^]^ to the increased milk iron by IONPs. Differences in the results were most noticeable for milk cells after IONP treatment (2.8 mg Fe kg^–1^) contributing 96.3% of increased milk iron, but not for FeC. In parallel to IONP dose‐dependent changes of total milk iron, milk cell iron also showed a significant increase response to IONPs in a Lognormal dose‐dependent manner, indicating the predominant roles of milk cells in delivering iron after IONP treatment and having their upper bounds (Figure 4f). These results demonstrated a novel iron delivery pathway in the mammary gland triggered by IONPs.

As milk cells are a cluster of various cell types,^[^ ^30^ ^]^ we further depicted IONP‐induced milk cell profiles and the sub‐distribution of milk cell iron in each cell type (Figure 4g–i). IONPs increased the percentage of neutrophils and macrophages in milk cells, and decreased the MEC percentage compared with the control and FeC group. The increased leukocyte percentage was not resulted from the mammary inflammation because the milk somatic cell count or blood immune index was not changed (*p* > 0.05, Figures S3 and S4, Supporting Information) and the immune infiltration was not significant in the mammary tissue (Figure S5, Supporting Information). The results inferred the IONP‐triggered migration of mammary‐resident leukocytes into milk. Indeed, breast milk leukocytes are thought to protect the mammary gland from infection during lactation.^[^ ^31^, ^32^, ^33^ ^]^ Interestingly, macrophages contributed the majority of milk iron (40.9%) in total milk cells under the IONP treatment, despite their minor percentages (5.0%). This may be benefitted from their functions of mainly capturing the nanoparticles during their mammary retention periods, which are similar to the Kupffer cells in the liver, followed by the migration of IONPs trapping macrophages into the milk. Neutrophils exerted similar functions as macrophages, but showed weaker ability to transfer the IONPs or IONP derivates into milk.

The predominant roles of mammary leukocytes (especially macrophages) on targeted IONP‐derived iron transfer into milk were further investigated using the macrophage‐depletion method in vivo. The clodronate liposomes are commonly used to deplete macrophages.^[^ ^34^, ^35^, ^36^ ^]^ In our study, the macrophages in the circulatory system and mammary gland of lactating mice were successfully exhausted within 72 h after treating with clodronate liposomes intraperitoneally and were gradually recovered after 72 h macrophage depletion treatment (Figure S6, Supporting Information). This efficiency measure of macrophage depletion ensured the reasonable time range (within 72 h) for subsequent experiments (Figure S6a,b, Supporting Information). The IONP, FeC, or PBS administration was performed on lactating mice 24 h after clodronate liposome injection as macrophage^–/–^ group or PBS injection as wild‐type group. Milk iron content in the IONP‐treated macrophages^–/–^ group was significantly lower than that in IONP‐treated wild‐type group, except for day 3 when macrophage gradually recovered (Figure S6c, Supporting Information). However, the macrophage recovery leads to a relative lower milk iron concentration than IONP‐treated wild‐type group in following days, which could be attributed to that largely finished nanoparticle removal by other cells or other organs from circulation during the macrophage depletion period in the IONP‐treated macrophages^–/–^ group. As no significant difference was observed between untreated macrophage^–/–^ group and wild‐type group or between FeC‐treated macrophage^–/–^ group and wild‐type group, the macrophage depletion showed little influence on physiological mammary iron transfer which is led by MEC secretion. These results revealed that the macrophage is indispensable for milk iron enhancement under IONP but not FeC administration.

The above evidences provided a functional dependence of macrophage on IONP‐induced milk iron enhancement. Since that macrophages capture nanoparticles have been widely investigated,^[^ ^37^ ^]^ we also observed similar evidences in mammary‐resident macrophages in the current study (Figure 3). Thus, IONP‐induced milk iron enhancement may additionally depend on macrophage migration into milk, subsequent to macrophage nanoparticle capture. In order to clarify this speculation, sinomenine hydrochloride, a well‐researched inhibitor of macrophage migration,^[^ ^36^ ^]^ was applied to lactating mice (Figure S7, Supporting Information). The efficiency of macrophage migration inhibition (MMI) was measured and ensured the effective time range as 24–72 h after sinomenine hydrochloride injection (Figure S7a, Supporting Information). In a pre‐established MMI model, the IONP, FeC, or PBS administration was performed on lactating mice (lactation day 1) 24 h after sinomenine hydrochloride injection as MMI group or PBS injection as wild‐type group (Figure S7b, Supporting Information). The MMI did not influence physiological mammary iron transfer as milk iron was enhanced by FeC in either MMI mice or wild‐type mice, and as milk iron had no significant difference between PBS‐treated MMI mice or wild‐type mice. During efficient MMI period, MMI‐animals showed significant lower milk iron concentrations than wild‐type mice after IONP treatment. When MMI efficiency was gradually disappeared, the milk iron level of IONP‐treated MMI group was recovered to a level statistically approaching to that of IONP‐treated wild‐type mice (*p* > 0.05). In a model of MMI post iron supplements, sinomenine hydrochloride or PBS was injected 72 h after the IONP, FeC, or PBS administration (Figure S7c, Supporting Information). The milk iron level of IONP‐treated group decreased to that of FeC‐treated group 24–48 h after MMI, and was gradually recovered 72 h after MMI when MMI efficiency was disappeared. These results revealed that the macrophage migration is indispensable for milk iron enhancement subsequent to macrophage nanoparticle capture under IONP but not FeC administration.

The milk cells were alive and sequestered iron intracellularly indicated by the 4’,6‐diamidino‐2‐phenylindole (DAPI) and iron tracer staining (Figure S8a, Supporting Information). In detail, the intracellularly sequestrated iron was mainly non‐IONP forms as IONPs were low in main mammary iron carriers including macrophages (less than 0.05% of total milk iron) and neutrophils (less than 0.01% of total milk iron, Figure S8b, Supporting Information). A trace amount of iron (0.16–0.60% secreted from milk cells, Figure S8c, Supporting Information) in the medium culturing the milk cell (1.36 ×10^6^ cell) in vitro further demonstrated the persistent iron sequestrations. The iron released by macrophages or neutrophils only counted for 0.07–0.12% and 0.02–0.03% of total iron in milk cells, respectively, in control or FeC group (Figure S8d, Supporting Information). Iron release abilities of macrophages or neutrophils in IONP group were not significantly elevated and also exhibited a comparable 0.06–0.11% and 0.02–0.03% of total iron in milk cells, respectively. Thus, the novel iron delivery pathway serves as a “cell cage”‐like pathway, which has never been revealed but once speculated and simulatively calculated by other,^[^ ^29^ ^]^ to avoid excessive extracellular free iron. This pathway has its advantages because free extracellular iron promotes invading pathogens. Moreover, the captured IONP‐iron by leukocytes may in turn promote both innate and adaptive immunity.^[^ ^38^ ^]^ As studies have shown, intracellular iron is proved to activate NF‐*κ*B via promoting the release of reactive oxygen species,^[^ ^39^ ^]^ and activate hypoxia‐inducible factor‐1 alpha (HIF‐1*α*) to promote the production of antimicrobial peptides by macrophages.^[^ ^40^, ^41^ ^]^ These results are also parallel to our findings that milk macrophage percentage increased in IONP‐treated lactating mice (Figure 4h).

### 2.3 Potential Therapeutic Effect of IONPs on Neonatal Anemia

**Figure 5:**
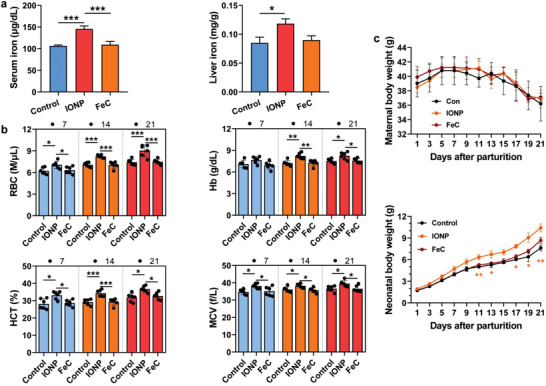
Treatment effect of IONPs as an iron supplement on a neonatal anemia model. a) Serum and liver iron status in a neonatal anemia mouse model affected by maternal IONP, FeC, or PBS treatment on day 21. b) Neonatal iron homeostasis indicated by RBC levels, hemoglobin (Hb), and HCT values, and MCV values and affected by maternal IONP, FeC, or PBS treatment on day 7, 14, 21 after birth in a neonatal anemia mouse model. c) Maternal body weight (upper) and neonatal body weight (lower) were recorded every other day during the whole lactation under the maternal intravenous treatment of IONPs, FeC, or PBS. The data are presented as the mean ± standard deviation (*n* = 6; for neonatal body weight measurement *n* represents a litter). *, **, and *** denote statistical significances, *p* < 0.05, *p* < 0.01, and *p* < 0.001, respectively.

As IONPs enhanced milk iron through their special pathways, we thus evaluate whether maternal IONP supplement show therapeutic effects on their anemic neonates (**Figure** **5**). To model preterm infants with iron restriction, pregnant C57BL/6J mice during E7.5‐17.5 were injected subcutaneously with 10 nmol of minihepcidin (PR73) dissolved in SL220 (Figure S9, Supporting Information).^[^ ^42^, ^43^, ^44^ ^]^ This dose of hepcidin agonist did not cause maternal anemia but caused neonatal anemia, tissue iron deficiency, and decreased weight.^[^ ^43^, ^44^, ^45^ ^]^ Then, preterm mother received a single intravenous injection of iron supplements (IONP, FeC) at a clinical iron level (2.8 mg Fe kg^–1^) or PBS. Maternal IONP treatment caused increased liver iron storage (39.2%), serum iron levels (by 37.0%), in breast‐fed offspring, but FeC and PBS did not (*p* > 0.05), indicating that maternal IONP treatment exerted iron transfer into preterm offspring with anemia via breastfeeding (Figure 5a). For hematological parameters in anemia offspring (Figure 5b), neonates from IONP‐treated group had the most efficient rescue in offspring anemia, with significantly increased red blood cell (RBC) count, hemoglobin, hematocrit (HCT), and mean corpuscular volume (MCV) (*p* < 0.05).

We further confirmed that breastfeeding by maternal IONP treatment did not induce inflammation or toxicity, as reflected by the stable liver and intestinal serum amyloid A1 (*Saa1*) mRNA (*p* > 0.05, Figure S10, Supporting Information). To evaluate the erythropoietic response, we measured mRNA expression of offspring bone marrow glycophorin A (*Gypa*) and erythroferrone (*Erfe*) (Figure S10, Supporting Information). Offspring *Erfe* and *Gypa* were both significantly elevated in IONP groups (*p* < 0.05), suggesting an effectual erythropoietic response to anemia via maternal iron transfer.

Neonatal anemia is associated with stillbirths or decreased birth weight.^[^ ^46^ ^]^ To assess the role of breastfeeding iron delivery on postnatal growth, body weight was monitored in pups breastfed by IONP, FeC, or PBS‐treated mothers. The growth (body weight) was significantly elevated in these anemia pups nursing from IONP‐treated mothers compared with pups nursing from FeC‐ or PBS‐treated dams (Figure 5c). These results confirmed the potential therapeutic effects of maternal IONP supplement on anemic offspring.

### 2.4 Potential Therapeutic Effect without Infection Risks of IONPs on Neonatal Anemia

The oral iron supplement, including iron fortified formula milk, has been widely studied in infants and children.^[^ ^47^ ^]^ In most conditions, the oral iron supplement has been shown to reduce anemia.^[^ ^48^ ^]^ However, side effects of oral iron supplement have been reported such as diarrhea and intestinal inflammation under conditions of disease burden.^[^ ^49^ ^]^ Evidences infer that the effects of orally supplemental iron on the gut disease or intestinal inflammation in infants are more pronounced in conditions where hygiene standards are low and the microbiome is susceptible to enteropathogens.^[^ ^50^ ^]^ The reason lies in large increases in intestinal iron by oral administration. For most enteropathogens (*Salmonella*, *Shigella*, or pathogenic *E. coli*), iron acquisition plays an essential role in virulence and colonization.^[^ ^51^ ^]^ By contrast, *lactobacilli*, a major group of beneficial “barrier” bacteria improving gut integrity and reducing colonization by enteropathogens, do not require iron.^[^ ^52^ ^]^ Therefore, an increase in intestinal iron can favor growth of enteropathogens over “barrier” bacteria. Thus, safe strategies of oral iron supplementation need to be improved such as reducing oral iron dosage while maximizing absorption to retain efficacy and sequestrating iron from enteropathogenic utilization. Unfortunately, there is no comparable system for sequestration of dietary iron known in the gut lumen.

**Figure 6:**
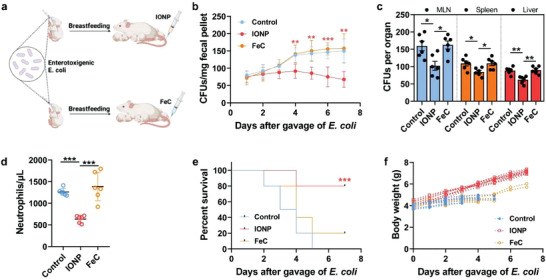
Treatment effect of IONPs as a maternal iron supplement on their breastfed neonates with late‐onset sepsis modeled by gut‐residing *Escherichia coli* (*E. coli*). a) Scheme of neonatal late‐onset sepsis modeled by gut‐residing *E. coli* and the treatment of IONPs, FeC, or PBS (as a control). 2 × 10^5^ CFUs of *E. coli* were gavaged to neonatal mice on day 7 after birth in conventionally rearing. b) CFUs of *E. coli* in breastfed neonatal stool following maternal administration of IONPs, FeC, or PBS. c) *E. coli* CFUs of MLN, spleen, and liver in neonates with late‐onset sepsis 3 days following maternal administration of IONPs, FeC, or PBS. d) Neutrophils in the neonatal blood 48 h following gavage of 2 × 10^5^ CFUs of *E. coli* and breastfed from intravenous IONP‐, FeC‐, or PBS‐treated mothers. e) Survival of neonatal mice following gavage of 2 × 10^5^ CFUs of *E. coli* and breastfed from intravenous IONP‐, FeC‐, or PBS‐treated mothers. f) Body weight of neonatal mice following gavage of 2 × 10^5^ CFUs of *E. coli* and breastfed from intravenous IONP‐, FeC‐, or PBS‐treated mothers. Black dots denote moribund pups. The data are presented as the mean ± standard deviation. *n* = 6 individuals in each group in (b)–(d). *n* = 10 in each group in (e). *n* = 10 mice per group in (f). *, **, and *** denote statistical significances, *p* < 0.05, *p* < 0.01, and *p* < 0.001, respectively.

We thus consider whether the novel pathway‐predominated iron delivery of IONPs not only exerts the iron supplements for infant, but also protects the milk iron from providing a hotbed for bacteria and plays a role of killing bacteria. Based on the preterm mouse pups, we developed a model of late‐onset neonatal sepsis, which is a leading cause of neonatal mortality affected by gut‐originating pathogen *E. coli*. Late‐onset sepsis (LOS) is a highly consequential complication of preterm birth and results in the rapid sepsis‐like death of neonates after bacterial translocation.^[^ ^53^ ^]^ Infant suffering from LOS is also lack of iron storage but have infection risks when supplied with iron.^[^ ^54^ ^]^ Mouse pups were colonized with *E. coli* by oral gavage of 2 × 10^5^ colony‐forming units (CFUs) (**Figure** **6**a). *E. coli* strain was found in the stool of the pups for at least 7 days following gavage confirming colonization (Figure 6b). The colonized *E. coli* was gradually suppressed in pups breastfed by IONP‐treated mothers (2.8 mg Fe kg^–1^). *E. coli* could be found in the mesenteric lymph node (MLN), spleen, and liver of all mice 3 days after gavage, but pups treated with IONP‐milk contain lowest *E. coli* in these organs (decreased by 22.2–35.9% compared to control group, *p* < 0.05, Figure 6c). *E. coli*‐treated pups developed neutrophilia (Figure 6d) and had a high death rate (Figure 6e), and were characterized illness by a lack of weight gain (Figure 6f) and lethargy. This lethality of *E. coli* to pups can be suppressed by breastfeeding from IONP‐treated mothers (*p* < 0.01), whereas oral iron supplements failed to suppress the lethality of *E. coli* to pups.

**Figure 7:**
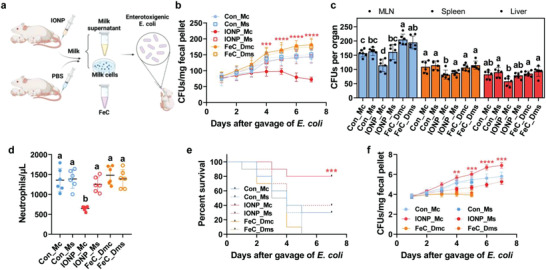
Effect of cells or supernatant from milk of lactating mice with/without IONPs on neonatal late‐onset sepsis. a) Scheme of neonatal late‐onset sepsis modeled by gut‐residing *Escherichia coli* (*E. coli*) and treatments of directly oral FeC mimicking the equivalent iron supplied by milk cells (FeC_Dmc, 10.3 mg Fe mL^–1^) and milk supernatant (FeC_Dms, 5.8 mg Fe mL^–1^) from IONP‐treated mothers, and cells or supernatant from milk of lactating mice treated with IONPs, or PBS (as a control). 2 × 10^5^ CFUs of *E. coli* were gavaged to neonates on day 7 after birth in conventionally rearing. b) CFUs of *E. coli* in neonatal stool following above administrations in (a). c) *E. coli* CFUs of MLN, spleen, and liver in neonates with late‐onset sepsis 3 days following above administrations in (a). Same letters mean no significant difference, different letters (“a,” “b,” “c”) mean significant difference. d) Neutrophils in the neonatal blood 48 h following gavage of *E. coli* and above administrations in (a). Same letters mean no significant difference, different letters (“a,” “b”) mean significant difference. e) Survival of neonatal mice following gavage of *E. coli* and above administrations in (a). f) Body weight of neonatal mice following gavage of *E. coli* and above administrations in (a). The data are presented as the mean ± standard deviation. *n* = 6 in each group in (b)–(d). *n* = 10 in each group in (e) and (f). *, **, and *** denote statistical significances, *p* < 0.05, *p* < 0.01, and *p* < 0.001, respectively.

To illuminate roles of endogenous leukocyte‐assisted pathway in treating anemic neonates with infection, the milk was divided into milk cells leading by endogenous leukocyte‐assisted pathway, and milk supernatants leading by general mammary delivery pathway (**Figure** **7**a). The iron transferred by endogenous leukocyte‐assisted pathway is strictly sequestered within cells and prevented from being utilized by pathogens on the gut mucosa, but can be utilized by host cells through milk cell homing into specific offspring tissue such as liver (Figure S8e, Supporting Information).^[^ ^55^ ^]^ In contrast, iron transferred by general mammary pathways are mainly presented in milk supernatants, and are accessible to pathogens for proliferations.^[^ ^56^ ^]^ The new born pups were separated into six groups and perform *E. coli* colonization as described above. For first two groups, milk cells and milk supernatant from IONP‐treated mothers (2.8 mg Fe kg^–1^) were gavaged, respectively, to each group. The control groups were set as pups gavaged by milk cells and milk supernatant from PBS‐treated mothers. The milk cells and milk supernatant from FeC‐treated mothers were not designed because maternal FeC treatment did not significantly alter the maternal iron transfer (see above) and was deemed as an insufficient way of iron supply for pups. Accordingly, direct oral FeC gavage to pups was designed to investigate whether direct iron supply can be an efficient and healthy way for iron supplement. The directly oral FeC treatment was set as two doses mimicking the equivalent iron supplied by milk cells in PBS (10.3 mg Fe mL^–1^), and milk supernatant (5.8 mg Fe mL^–1^) from IONP‐treated mothers.

Only milk cells from maternal IONP treatment suppressed the *E. coli* colonization (*p* < 0.01), whereas *E. coli* colonization were not prevented by milk cells and milk supernatant from maternal PBS‐treated group, the direct iron treatment even increased the *E. coli* colonization (Figure 7b,c). Neutrophilia was developed by *E. coli* colonization in groups treated with milk cells and supernatant from maternal PBS‐treated group, milk supernatant from maternal IONP‐treated group, and direct iron treatment groups, but was relieved by milk cells from maternal IONP treatment (Figure 7d). The mean lethality rate per litter in mice treated with milk cells from maternal PBS‐treated group, milk supernatant from IONP‐, PBS‐treated group, and direct iron treatment group exposed to *E. coli* was 60–100% (Figure 7e). The milk cells from maternal IONP treatment had a lower lethality with 20%, and showed a beneficial effect on neonatal intestine (Figure S11, Supporting Information). Moreover, the loss of weight in anemic neonates with *E. coli* colonization was reversed by milk cells derived from milk of IONP‐treated lactating mice (*p* < 0.01, Figure 7f). Thus, following treatment of milk cells from maternal IONP treatment in pups, *E. coli* was prevented from colonization, gut dissemination, and virulence exertion.

## 3 Conclusion

This work demonstrates a novel nutrient deliver pathway in the lactating mammary glands triggered by IONPs, contributing to predominantly increased milk iron in IONP treatments. In this pathway, endogenous leukocytes including macrophages and neutrophils play predominant roles, showing IONP transformation into iron ion, iron delivery, and iron segregation during the maternal IONP treatment. This pathway serves as “cell cage”‐like iron supplements preventing the pathogen iron utilization and thus decreasing the neonatal infections during the iron supplement. Our findings provide a promising therapeutic paradigm to effectively and safely supply iron for anemic neonate by maternal IONP treatment at a clinical dose, which is also potentially available for other nutrients at nanoscale.

## 4 Experimental Section

### Preparation and Physicochemical Characterization of IONPs

IONPs (Fe_3_O_4_ nanoparticles; purity: >99%) were prepared chemically via a co‐precipitation method.^[^ ^57^ ^]^ In a typical experiment, 4/3 mmol of FeCl_3_ and 2/3 mmol of FeCl_2_, with the ratio of ferric ion to ferrous ion in the solution being 2, were dissolved in 100 mL of deionized water under a nitrogen gas flow. At the same time, 5 g of sodium citrate was added into the solution. Then, this mixed solution was stirred and added with excess NH_4_OH or NaOH (two to three times) while the temperature was kept constant at 80 ℃. The reaction of precipitates was carried out under high‐speed stirring. The mixture was cooled to room temperature and centrifuged. The precipitate was washed twice with deionized water and finally re‐dispersed into deionized water.

The morphology and diameter of IONPs were determined by transmission electron microscopy (TEM) using FEI Tecnai F20 transmission electron microscope. The size distribution spectrum, and zeta potential of IONPs were measured by a Zetasizer Nano‐ZS (Malvern Instruments, Worcestershire, UK) after the IONP were suspended (10 mg mL^−1^) in double deionized water and dispersed. The specific surface area of IONPs was calculated by formula of average weight specific surface area

(1) $$S_{W}=\frac{3}{\rho r}\;$$

where *S*_W_ means the average weight‐specific surface area, *ρ* is the density of material, and *r* represents the average particle size of nanoparticles.

### Maternal Iron Supplement Treatment and Modeling

In this study, the intravenous dose of IONP and FeC treated in mice was chosen as 2.8 mg Fe kg^–1^ body weight, which is potentially used in human clinic.^[^ ^23^, ^58^, ^59^ ^]^ For short‐term iron distribution or biotransformation study, the blood and milk were sampled at different time‐points after injection (hours, h0.25, h0.5, h0.75, h1, h2, h3, h4, h6, h8, h10, h12, h16, h20, and h24; sampling volume: 20 µL per time point). For long‐term iron distribution or biotransformation study, mice were sacrificed at different time‐points after injection (days, D1, D3, D7, D11, D14, and D21). Liver, spleen, kidney, and mammary gland were excised and shared for ICP‐MS, FMR, and TEM characterizations. Mice sampled with injury were not used for subsequent tests.

For macrophage depletion model, clodronate liposome was intravenously injected in lactating mice to deplete circulatory and mammary‐resident macrophages using the adjusted dose according to the manufacturer's instructions (0.15–0.2 mL/25 g).**^[^** ^36^, ^60^ **^]^** The control liposome was used to evaluate and eliminate the effect from liposome. The efficiency of macrophage depletion was confirmed at 24, 48, and 72 h after the clodronate liposome injection by examining the number of mammary‐resident macrophages by flow cytometry and blood monocytes by complete blood counts. Clodronate liposome (CAS: 40337ES08) and control liposome (CAS: 40338ES05) were purchased from Yeasen Biotechnology (Shanghai, China).

For MMI model, sinomenine hydrochloride (Macklin, Shanghai, China; CAS: 115‐53‐7; purity: >97%) injection was performed intraperitoneally (50 mg kg^−1^).^[^ ^36^ ^]^ Control group was intraperitoneally injected with PBS. The inhibitory efficiency of macrophage migration into milk was verified at 24, 48, and 72 h after the sinomenine hydrochloride injection by examining the number of milk macrophage by flow cytometry.

Animal procedures and protocols were performed in accordance with the Institutional Animal Care and Use Committee at Zhejiang University (20041).

### Iron Quantification

Total iron concentrations in samples were determined by ICP‐MS. The samples were mineralized in nitric acid and chloridric acid (7:3) for 4 h at 100 °C and then treated with H_2_O_2_ for 1 h at 95 °C on a heat block. The sample was adjusted to a volume of 10 mL with distilled water and was then determined for total iron concentration by ICP‐MS (PerkinElmer NexION 300X, USA). Plasma iron turnover was determined according to a previous study.^[^ ^61^ ^]^

IONPs were quantified by a Varian E102 Electron Paramagnetic Resonance spectrometer operating at Q band (9.26 GHz). The first derivative of the power absorption *dW*(*B*)/*dB* was obtained as a function of the applied field *B* (0–6000 Gauss). The modulation field had a frequency of 100 kHz and an amplitude of 10 G. The amount of IONPs in the sample was calculated by the area under the FMR absorption curve and a calibration curve obtained by suspensions of IONPs at different concentrations quantified by ICP‐MS. Before FMR analysis, samples were weighed, rinsed, sliced, and dried. The dried samples were weighed again and put into a quartz tube for FMR analysis.

### TEM

Tissue samples were collected, cut into 1 mm^3^ pieces, and fixed with 2.5% glutaraldehyde and further fixed with 1% osmium tetroxide containing 1.5% potassium cyanoferrate, contrasted with 2% uranyl acetate. The samples were then dehydrated with a graded series of ethanol solutions (30–100%) and embedded in Epon. Ultrathin sections (70 nm) were collected from samples onto copper grids and counterstained with lead citrate. The observation of the samples was performed by a TEM (Hitachi Model H‐7650, Hitachi, Japan).^[^ ^62^ ^]^ The energy‐dispersive X‐ray spectroscopy (EDX) was performed to generate the iron chemical map on a Technai G2F20 field‐emission electron microscope equipped with an energy‐dispersive X‐ray analyzer.

### Hematoxylin–Eosin (HE) Staining

The collected samples were fixed in a 4% paraformaldehyde solution for 4 h, imbedded in paraffin, sectioned into 5 µm, deparaffinized and hydrated, and stained with hematoxylin and eosin.^[^ ^63^ ^]^

### Immunofluorescence Analysis

The sectioned slices were prepared as described in “HE staining.” The slices were permeabilized with PBS containing 0.3% Triton X‐100, and incubated with blocking buffer, and incubated with primary antibody (Anti‐GFP, Abcam), followed by Alexa Fluor 488‐conjugated secondary antibody (Abcam). The slices were counterstained by DAPI (Vector Laboratories).^[^ ^64^ ^]^

### Localization of Iron in Milk

The collected milk was centrifuged for 15 min (200 × *g*, 4 °C) to separate milk cells (precipitate) and cell‐free milk (supernatant). The iron contents in milk cells and cell‐free milk were determined by ICP‐MS as described above. For further analysis of milk cell profile and iron distribution in each milk cell type, flow cytometry sorting was used.^[^ ^62^ ^]^ The precipitate obtained from milk centrifugation was collected and incubated with fluorescent‐labeled antibodies CD24, CD45, CD11b, Ly6G, and F4/80 for 15 min at 25 °C (Table S1, Supporting Information). Milk cells were then washed in PBS and incubated with DAPI for 15 min at 25 °C (Table S1, Supporting Information). Milk cells were analyzed and sorted with a Flow Cytometry Sorter (Becton Dickinson, USA; gating strategy seen in Figure S12, Supporting Information). The background fluorescence was standardized by respective isotype controls (Table S1, Supporting Information). The sorted milk cells were pooled according to groups and cell types and used to quantify the iron content by ICP‐MS. For analysis of iron in each milk component, milk lipids were obtained from the lipid layer of cell‐free milk after centrifugation for 30 min (3000 × *g*, 4 °C). The skimmed cell‐free milk was centrifuged at 50 000 × *g* (4 °C) for 30 min and ultrafiltered in a Minicon cell with a membrane molecular weight cut‐off of 15 kDa, to separate low molecular weight compounds. The protein fractions of milk were studied by gel filtration of skimmed milk on a Sephadex G‐200 column (2.6 × 80 cm). The column was equilibrated with 0.1 m ammonium acetate buffer (pH: 6.9), and a flow rate of 12 mL h^–1^ was used. Protein was assayed spectrophotometrically at 280 nm. Iron in these milk components was determined by ICP‐MS.

### Complete Blood Counts

Blood was collected from mice by submandibular vein puncture into K2EDTA containing tubes, blood counts were performed on a hematology autoanalyzer (IDEXX Laboratories, USA).

### Modeling of Preterm and Anemia Offspring

PR73 was used for modeling of preterm and anemic offspring. PR73 was dissolved in 80% ethanol and then mixed with 60 mg of SL220 (NOF). The ethanol was evaporated off using a vacuum chamber warmed to 50 ℃. The resultant gel was stored up to 24 h at 4 ℃ and re‐dissolved in water. Pregnant C57BL/6J mice during E7.5‐17.5 were injected subcutaneously with 10 nmol of PR73.^[^ ^43^ ^]^

### Quantitative Polymerase Chain Reaction (qPCR)

RNA was prepared using TRIzol reagent according to the manufacturer's instructions (Invitrogen). The cDNA was synthesized using iScript cDNA Synthesis Kit (Bio‐Rad). qPCR was performed with SsoAdvanced SYBR Green Supermix (Bio‐Rad) and primers (Table S2, Supporting Information), and samples were run in duplicate on a CFXconnect instrument (BioRad).

### *E. coli* Infection

On day 7 of birth, mice were gavaged with 20 µL PBS containing 10^7^ CFUs mL^–1^ (2 × 10^5^ CFUs) *E. coli*. Enterotoxigenic *E. coli* was obtained from BioVector NTCC. Cohoused littermates were used when possible to minimize differences in the original gut microbiota. Stool was collected to observe colonization. Health status of mice, including lack of weight gain, lethargy, inflammation, and death, was monitored. The translocation of enteric bacteria to extraintestinal organs (spleen, MLN, and liver) was monitored by comparing the multilocus sequence type (MLST) of recovered colonies to those of the challenge *E. coli*.^[^ ^53^ ^]^ Moribund was identified as lethargic neonates that remained motionless and had a growth retardation. In survival experiments, neonates were monitored daily for signs of disease, weight gain, and death. In some neonates, 3 days following gavage and experimental treatment, spleen, MLN, and liver were isolated to analyze bacterial translocation.

### Bacterial Quantification in Organs and Stool

Spleen, MLN, liver, or stool were homogenized in PBS by 0.1 mm diameter zirconium silica beads (BioSpec) and homogenized Bullet Blender Tissue Homogenizer (Next Advance). The obtained homogenate was quantified for *E. coli* strain by MLST identification.^[^ ^53^ ^]^

### Statistical Analysis

Data were analyzed using GraphPad Prism 8.0 (GraphPad Software, Inc., San Diego, CA, USA) and all results were presented as means ± standard deviation. Outliers were evaluated using the ROUT method (*Q* = 1%). One‐way analysis of variance was used to analyze the statistical differences between three or more groups with Tukey post hoc test. For two groups analysis, two‐tailed Student's *t* test was used. For survival analysis, Kaplan–Meier curves were used with the log‐rank test. In all cases, values with *p* < 0.05 were considered as statistically significant. The number of animals or repeated experiments is presented in each figure legend.

## Conflict of Interest

The authors declare no conflict of interest.

## Supporting information

Supporting Information

## Data Availability

The data that support the findings of this study are available from the corresponding author upon reasonable request.

## References

[1] K. E. Lyons , C. A. Ryan , E. M. Dempsey , R. P. Ross , C. Stanton , Nutrients 2020, 12, 1039. 10.3390/nu12041039 PMC7231147 32283875

[2] J. Cai , D. Wang , J. Liu , J. Sci. Food Agric. 2018, 98, 1261. 28758674 10.1002/jsfa.8605

[3] J. L. McManaman , M. C. Neville , Adv. Drug Delivery Rev. 2003, 55, 629. 10.1016/s0169-409x(03)00033-4 12706546

[4] M. Keikha , M. Bahreynian , M. Saleki , R. Kelishadi , Breastfeed. Med. 2017, 12, 517. 28880568 10.1089/bfm.2017.0048

[5] F. Bravi , F. Wiens , A. Decarli , A. D. Pont , C. Agostoni , M. Ferraroni , Am. J. Clin. Nutr. 2016, 104, 646. 27534637 10.3945/ajcn.115.120881

[6] E. Sosa‐Castillo , M. Rodríguez‐Cruz , C. Moltó‐Puigmartí , Br. J. Nutr. 2017, 118, 161. 28831952 10.1017/S0007114517001854

[7] C. J. Bautista , L. A. Reyes‐Castro , R. J. Bautista , V. Ramirez , A. L. Elias‐López , R. Hernández‐Pando , E. Zambrano , Reprod. Sci. 2021, 28, 2481. 34159572 10.1007/s43032-021-00492-8

[8] L. Daniels , R. S. Gibson , A. Diana , J. J. Haszard , S. Rahmannia , D. E. Luftimas , D. Hampel , S. Shahab‐Ferdows , M. Reid , L. Melo , Y. Lamers , L. H. Allen , L. A. Houghton , Am. J. Clin. Nutr. 2019, 110, 391. 31152543 10.1093/ajcn/nqz047 PMC6669051

[9] S. Lee , S. L. Kelleher , Am. J. Physiol. Endocrinol. Metab. 2016, 311, E405. 27354238 10.1152/ajpendo.00495.2015 PMC5005964

[10] M. Domellöf , B. Lönnerdal , K. G. Dewey , R. J. Cohen , O. Hernell , Am. J. Clin. Nutr. 2004, 79, 111. 14684406 10.1093/ajcn/79.1.111

[11] M. Nakamori , N. X. Ninh , H. Isomura , N. Yoshiike , V. T. T. Hien , B. T. Nhug , N. V. Nhien , T. Nakano , N. C. Khan , S. Yamamoto , J. Nutr. Sci. Vitaminol. 2009, 55, 338. 19763035 10.3177/jnsv.55.338

[12] A. Bzikowska , A. Czerwonogrodzka‐Senczyna , A. Wesołowska , H. Weker , Pol. Merkuriusz Lek. 2017, 43, 276. 29298968

[13] R. S. Gibson , S. Rahmannia , A. Diana , C. Leong , J. J. Haszard , D. Hampel , M. Reid , J. Erhardt , A. H. Suryanto , W. N. Sofiah , A. Fathonah , S. Shahab‐Ferdows , L. H. Allen , L. A. Houghton , Am. J. Clin. Nutr. 2020, 112, 1039. 32844187 10.1093/ajcn/nqaa200 PMC7528569

[14] C. Y. Wang , J. L. Babitt , Blood 2019, 133, 18.30401708

[15] N. Montalbetti , M. G. Dalghi , C. Albrecht , M. A. Hediger , J. Mammary Gland Biol. Neoplasia 2014, 19, 73. 24567109 10.1007/s10911-014-9317-9

[16] B. Lönnerdal , Annu. Rev. Nutr. 2007, 27, 165. 17506666 10.1146/annurev.nutr.27.061406.093809

[17] N. D. Donahue , H. Acar , S. Wilhelm , Adv. Drug Delivery Rev. 2019, 143, 68. 10.1016/j.addr.2019.04.008 31022434

[18] S. Behzadi , V. Serpooshan , W. Tao , M. A. Hamaly , M. Y. Alkawareek , E. C. Dreaden , D. Brown , A. M. Alkilany , O. C. Farokhzad , M. Mahmoudi , Chem. Soc. Rev. 2017, 46, 4218. 28585944 10.1039/c6cs00636a PMC5593313

[19] H. Zou , J. Zhu , D. S. Huang , Expert Opin. Drug Delivery 2019, 16, 251. 10.1080/17425247.2019.1581762 30742557

[20] A. Parodi , N. Quattrocchi , A. L. Ven , C. Chiappini , M. Evangelopoulos , J. O. Martinez , B. S. Brown , S. Z. Khaled , I. K. Yazdi , M. V. Enzo , L. Isenhart , M. Ferrari , E. Tasciotti , Nat. Nanotechnol. 2013, 8, 61. 23241654 10.1038/nnano.2012.212 PMC3751189

[21] L. P. Jahromi , M. A. Shahbazi , A. Maleki , A. Azadi , H. A. Santos , Adv. Sci. 2021, 8, 2002499. 10.1002/advs.202002499 PMC8061401 33898169

[22] M. Ayer , H. A. Klok , J. Controlled Release 2017, 259, 92. 10.1016/j.jconrel.2017.01.048 28189629

[23] F. Vanobberghen , O. Lweno , A. Kuemmerle , K. D. Mwebi , P. Asilia , A. Issa , B. Simon , S. Mswata , S. Schmidlin , T. R. Glass , S. Abdulla , C. Daubenberger , M. Tanner , S. Meyer‐Monard , Lancet Global Health 2021, 9, e189. 33245866 10.1016/S2214-109X(20)30448-4

[24] E. Rogozińska , J. Daru , M. Nicolaides , C. Amezcua‐Prieto , S. Robinson , R. Wang , P. J. Godolphin , C. M. Saborido , J. Zamora , K. S. Khan , S. Thangaratinam , Lancet Haematol. 2021, 8, e503. 34171281 10.1016/S2352-3026(21)00137-X PMC7612251

[25] A. Pagani , A. Nai , L. Silvestri , C. Camaschella , Front. Physiol. 2019, 10, 1294. 31649559 10.3389/fphys.2019.01294 PMC6794341

[26] R. S. Zeidan , S. M. Han , C. Leeuwenburgh , R. Xiao , Ageing Res. Rev. 2021, 72, 101510. 34767974 10.1016/j.arr.2021.101510 PMC8620744

[27] L. Jiang , J. Wang , K. Wang , H. Wang , Q. Wu , C. Yang , Y. Yu , P. Ni , Y. Zhong , Z. Song , E. Xie , R. Hu , J. Min , F. Wang , Blood 2021, 138, 689. 33895792 10.1182/blood.2020008986 PMC8394904

[28] B. J. Crielaard , T. Lammers , S. Rivella , Nat. Rev. Drug Discovery 2017, 16, 400. 28154410 10.1038/nrd.2016.248 PMC5455971

[29] J. K. Friel , J. Pediatr. Gastroenterol. Nutr. 2017, 64, 339. 27496800 10.1097/MPG.0000000000001364

[30] M. Witkowska‐Zimny , E. Kaminska‐El‐Hassan , Cell. Mol. Biol. Lett. 2017, 22, 11. 28717367 10.1186/s11658-017-0042-4 PMC5508878

[31] C. M. Fetherston , C. S. Lee , P. E. Hartmann , Adv. Nutr. Res. 2001, 10, 167. 11795040 10.1007/978-1-4615-0661-4_8

[32] D. P. Wirt , L. T. Adkins , K. H. Palkowetz , F. C. Schmalstieg , A. S. Goldman , Cytometry 1992, 13, 282. 1533582 10.1002/cyto.990130310

[33] F. Hassiotou , D. T. Geddes , Adv. Nutr. 2015, 6, 267. 25979492 10.3945/an.114.007377 PMC4424778

[34] M. Gliem , A. K. Mausberg , J. I. Lee , I. Simiantonakis , N. van Rooijen , H. P. Hartung , S. Jander , Ann. Neurol. 2012, 71, 743. 22718543 10.1002/ana.23529

[35] P. Seiler , P. Aichele , B. Odermatt , H. Hengartner , R. M. Zinkernagel , R. A. Schwendener , Eur. J. Immunol. 1997, 27, 2626. 9368619 10.1002/eji.1830271023

[36] W. J. Gao , J. X. Liu , Y. Xie , P. Luo , Z. Q. Liu , L. Liu , H. Zhou , Pharmacol. Res. 2021, 167, 105513. 33617975 10.1016/j.phrs.2021.105513

[37] K. M. Tsoi , S. A. MacParland , X. Z. Ma , V. N. Spetzler , J. Echeverri , B. Ouyang , S. M. Fadel , E. A. Sykes , N. Goldaracena , J. M. Kaths , J. B. Conneely , B. A. Alman , M. Selzner , M. A. Ostrowski , O. A. Adeyi , A. Zilman , I. D. McGilvray , W. C. Chan , Nat. Mater. 2016, 15, 1212. 27525571 10.1038/nmat4718 PMC5132626

[38] K. Roemhild , F. Maltzahn , R. Weiskirchen , R. Knüchel , S. Stillfried , T. Lammers , Trends Pharmacol. Sci. 2021, 42, 640. 34090703 10.1016/j.tips.2021.05.001 PMC7611894

[39] L. Chen , S. Xiong , H. She , S. W. Lin , J. Wang , H. Tsukamoto , J. Biol. Chem. 2007, 282, 5582. 17172471 10.1074/jbc.M609273200

[40] C. Renassia , C. Peyssonnaux , Curr. Opin. Hematol. 2019, 26, 125. 30855332 10.1097/MOH.0000000000000494 PMC6467554

[41] A. F. McGettrick , L. A. J. O'Neill , Cell Metab. 2020, 32, 524. 32853548 10.1016/j.cmet.2020.08.002

[42] G. C. Preza , P. Ruchala , R. Pinon , E. Ramos , B. Qiao , M. A. Peralta , S. Sharma , A. Waring , T. Ganz , E. Nemeth , J. Clin. Invest. 2011, 121, 4880. 22045566 10.1172/JCI57693 PMC3225996

[43] V. Sangkhae , A. L. Fisher , K. J. Chua , P. Ruchala , T. Ganz , E. Nemeth , Blood 2020, 136, 2206. 32584957 10.1182/blood.2020005745 PMC7645983

[44] E. Ramos , P. Ruchala , J. B. Goodnough , L. Kautz , G. C. Preza , E. Nemeth , T. Ganz , Blood 2012, 120, 3829. 22990014 10.1182/blood-2012-07-440743 PMC3488893

[45] J. Arezes , G. Jung , V. Gabayan , E. Valore , P. Ruchala , P. A. Gulig , T. Ganz , E. Nemeth , Y. Bulut , Cell Host Microbe 2015, 17, 47. 25590758 10.1016/j.chom.2014.12.001 PMC4296238

[46] S. Iqbal , C. Ekmekcioglu , J. Matern.‐Fetal Neonat. Med. 2019, 32, 1528. 10.1080/14767058.2017.1406915 29207894

[47] V. Garcés , A. González , L. Sabio , C. M. Sánchez‐Arévalo , N. Gálvez , J. M. Dominguez‐Vera , Materials 2020, 13, 481. 10.3390/ma13020481 PMC7014100 31963902

[48] P. S. Suchdev , M. E. D. Jefferds , E. Ota , K. da Silva Lopes , L. M. De‐Regil , Cochrane Database Syst. Rev. 2020, 2, CD008959. 32107773 10.1002/14651858.CD008959.pub3 PMC7046492

[49] D. Paganini , M. A. Uyoga , M. B. Zimmermann , Nutrients 2016, 8, 494. 10.3390/nu8080494 PMC4997407 27529276

[50] T. Lee , T. Clavel , K. Smirnov , A. Schmidt , I. Lagkouvardos , A. Walker , M. Lucio , B. Michalke , P. Schmitt‐Kopplin , R. Fedorak , D. Haller , Gut 2017, 66, 863. 26848182 10.1136/gutjnl-2015-309940 PMC5531225

[51] H. Naikare , K. Palyada , R. Panciera , D. Marlow , A. Stintzi , Infect. Immun. 2006, 74, 5433. 16988218 10.1128/IAI.00052-06 PMC1594910

[52] E. D. Weinberg , Perspect. Biol. Med. 1997, 40, 578. 9269745 10.1353/pbm.1997.0072

[53] K. A. Knoop , P. E. Coughlin , A. N. Floyd , I. M. Ndao , C. Hall‐Moore , N. Shaikh , A. J. Gasparrini , B. Rusconi , M. Escobedo , M. Good , B. B. Warner , P. I. Tarr , R. D. Newberry , Proc. Natl. Acad. Sci. U. S. A. 2020, 117, 7941. 32179676 10.1073/pnas.1912022117 PMC7148560

[54] P. Visitchanakun , W. Saisorn , J. Wongphoom , P. Chatthanathon , N. Somboonna , S. Svasti , S. Fucharoen , A. Leelahavanichkul , Am. J. Physiol.: Gastrointest. Liver Physiol. 2020, 318, G966. 32308038 10.1152/ajpgi.00337.2019

[55] A. Laouar , Trends Immunol. 2020, 41, 225. 32057705 10.1016/j.it.2020.01.005

[56] M. Nairz , G. Weiss , Mol. Aspects Med. 2020, 75, 100864. 32461004 10.1016/j.mam.2020.100864

[57] S. Sun , H. Zeng , J. Am. Chem. Soc. 2002, 124, 8204. 12105897 10.1021/ja026501x

[58] M. Levy , N. Luciani , D. Alloyeau , D. Elgrabli , V. Deveaux , C. Pechoux , S. Chat , G. Wang , N. Vats , F. Gendron , C. Factor , S. Lotersztajn , A. Luciani , C. Wilhelm , F. Gazeau , Biomaterials 2011, 32, 3988. 21392823 10.1016/j.biomaterials.2011.02.031

[59] J. Daru , Lancet Global Health 2019, 7, e1597.31708133

[60] M. Bijnen , T. Josefs , I. Cuijpers , C. J. Maalsen , J. van de Gaar , M. Vroomen , E. Wijnands , S. S. Rensen , J. W. M. Greve , M. H. Hofker , E. A. L. Biessen , C. D. A. Stehouwer , C. G. Schalkwijk , K. Wouters , Gut 2018, 67, 1317. 29074725 10.1136/gutjnl-2016-313654

[61] D. Trinder , J. K. Olynyk , W. S. Sly , E. H. Morgan , Proc. Natl. Acad. Sci. U. S. A. 2002, 99, 5622. 11943867 10.1073/pnas.082112299 PMC122820

[62] J. Cai , X. Zang , Z. Wu , J. Liu , D. Wang , Environ. Int. 2019, 133, 105153. 31520958 10.1016/j.envint.2019.105153

[63] X. X. Dai , Y. Jiang , J. H. Gu , Z. Y. Jiang , Y. W. Wu , C. Yu , H. Yin , J. Zhang , Q. H. Shi , L. Shen , Q. Q. Sha , H. Y. Fan , Adv. Sci. 2021, 8, 2003636. 10.1002/advs.202003636 PMC8132151 34026442

[64] X. X. Dai , Z. Y. Jiang , Y. W. Wu , Q. Q. Sha , Y. Liu , J. Y. Ding , W. D. Xi , J. Li , H. Y. Fan , Cell Rep. 2021, 37, 110007. 34788619 10.1016/j.celrep.2021.110007

