# Supplementary material for: Mammary Leukocyte‐Assisted Nanoparticle Transport Enhances Targeted Milk Trace Mineral Delivery
Source: Adv Sci (Weinh). 2022 Jun 30;9(26):2200841. doi: 10.1002/advs.202200841 (PMC9475556; doi:10.1002/advs.202200841)
Supplement: Supplementary file 1 — Supporting Information [file ADVS-9-2200841-s001.pdf]

ADVANCED SCIENCE
Open Access

Supporting Information

for *Adv. Sci.*, DOI 10.1002/advs.202200841

Mammary Leukocyte-Assisted Nanoparticle Transport Enhances Targeted Milk Trace Mineral Delivery

Jie Cai, Jie Peng, Xinwei Zang, Juan Feng, Ruocheng Li, Peng Ren, Bingzhu Zheng, Jiaying Wang, Juan Wang, Mi Yan, Jianxin Liu, Renren Deng and Diming Wang**

Supporting Information

Mammary Leukocyte-Assisted Nanoparticle Transport Enhances Targeted Milk Trace Mineral Delivery

Jie Cai†, *Jie Peng*†, *Xinwei Zang*†, *Juan Feng*†, *Ruocheng Li, Peng Ren, Bingzhu Zheng, Jiaying Wang*, *Juan Wang*, *Mi Yan*, *Jianxin Liu*, *Renren Deng**, *Diming Wang**

Analysis of Intracellular and Extracellular Iron from Milk Cells

1.36×10^6 milk cells isolated from lactating mice treated with iron oxide nanoparticles (IONPs), ferric carboxymaltose (FeC), or phosphate buffer saline (PBS, as a control) were seeded on glass bottom culture dishes (MatTek P35G-1.5-14-C). Cells were grown for an additional 24 h. In the meantime, liquids in culture medium were sampled at 1, 2, 4, 8, 16, and 24 h after cell culture began for the determination of extracellular iron secreted by milk cells. Extracellular iron was measured by inductively coupled plasma-mass spectrometry. Then, cells were stained in 1 μM Ferro orange (dojingo) in HBSS for 30 min and observed immediately for intracellular iron determination in milk cells. Focal images were obtained by a Zeiss inverted LSM 780 laser scanning confocal microscope (Zeiss) with the Cy3 filter (ex 514nm, em 525-596).

Immunofluorescence TUNEL Apoptosis Analysis

The sectioned slices of mammary tissues were prepared as described in "HE staining". The dewaxed sections were restored by proteinase K at 37 °C for 25 min and washed with PBS 3 times (5 min each). The sections were incubated with a membrane rupture liquid at 25 °C for 20 min and washed with PBS 3 times (5 min each). The apoptotic analysis of each section was performed by a TUNEL kit (Roche) with a mixture of terminal deoxynucleotidyl transferase and dUTP (mixed ratio was 1:9) at 37 °C for 2 h. Each section was counterstained with 4',6-diamidino-2-phenylindole (DAPI).

Western Blot Analysis

Liver samples were lysed and heated for 5 min at 95 °C. Protein lysates were separated through sodium dodecyl sulfate polyacrylamide gel electrophoresis and electrophoretically transferred to polyvinylidene fluoride membranes (Millipore), and blocked in Tris Buffered Saline Tween (TBST) containing 5% skim milk (Becton, Dickinson and Company) for 30 min. After

treatment with primary anti-bodies (Smad5, 1:200, Santa Cruz Biotechnology; phosphorylated Smad1/5/8, 1:500, Cell Signaling Technology; Hepcidin, 1:200, Abcam; Ferroportin, 1:200, Abcam), the membranes were washed with TBST, and incubated with HRP-linked secondary antibodies (Abcam) for 1 h, followed by three washes in TBST. The targeted proteins were observed by the Super Signal West Femto maximum sensitivity substrate (Thermo Fisher).

Table S1. Antibodies used for flow cytometry.

Antibody	Company	Category number	Dose
BB515 Rat Anti-Mouse CD45	Becton Dickinson	564590	1:100
APC Rat Anti-Mouse CD11B	Becton Dickinson	553312	1:100
PerCP-Cy5.5 Rat Anti-Mouse LY6G	Becton Dickinson	560602	1:100
PE-Cy7 Rat Anti-Mouse CD24	Becton Dickinson	560536	1:100
PE Rat Anti-Mouse F4/80	Becton Dickinson	565410	1:100
DAPI	Becton Dickinson	564907	1:20000
BB515 Rat IgG2b, κ Isotype Control	Becton Dickinson	564421	1:100
APC Rat IgG2b, κ Isotype Control	Becton Dickinson	553991	1:100
PerCP-Cy5.5 Rat IgG2a, κ Isotype Control	Becton Dickinson	550765	1:100
PE-Cy7 Rat IgG2b, κ Isotype Control	Becton Dickinson	552849	1:100
PE Rat IgG2a, κ Isotype Control	Becton Dickinson	553930	1:100

Table S2. Primers for qPCR.

	Fwd (5’->3’)	Rev (5’->3’)
Saa1	AGTCTGGGCTGCTGAGAAAA	ATGTCTGTTGGCTTCCTGGT
Gypa	ATGGCAGGGATTATCGGAAC	CACCCTCAGGAGATTGGATG
Erfe	ATGGGGCTGGAGAACAGC	TGGCATTGTCCAAGAAGACA

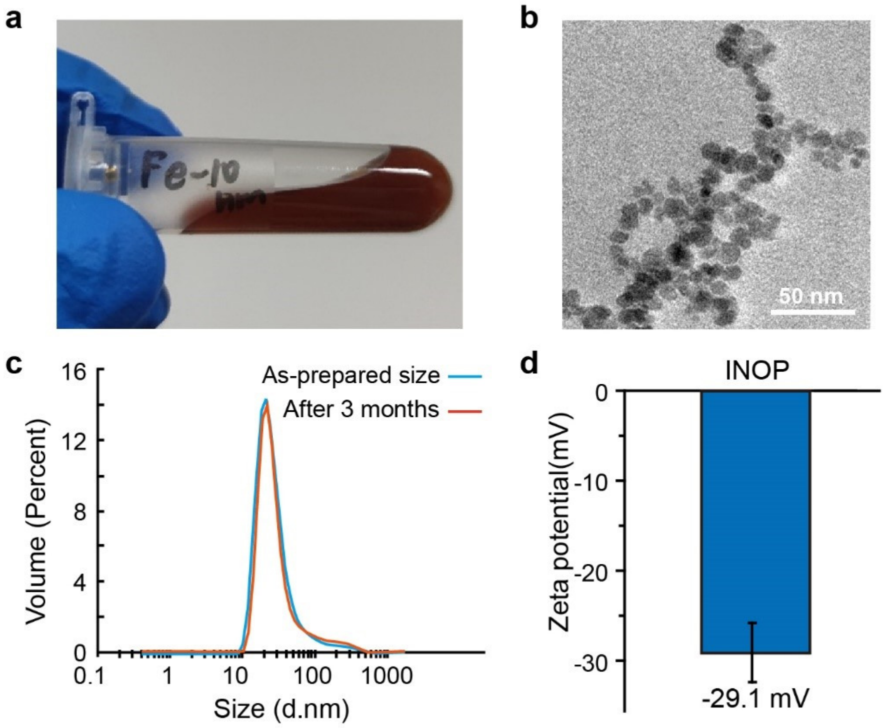

Figure S1. a, Photo of iron oxide nanoparticles (IONPs). **b**, TEM image of IONPs. **c**, Dynamic light scattering (DLS) measurement showing hydrodynamic particle size distribution of the same IONP sample (bule line represents as-prepared size, orange line represents the measurement after standing still for 3 months). **d**, The zeta potential of IONPs.

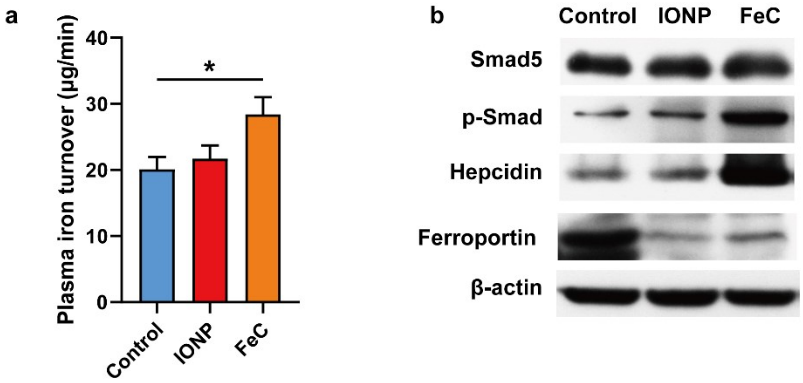

Figure S2. Systematic iron homeostasis influenced by iron supply. a, circulating transferrin-bound iron turnover in lactating mice with administration of iron oxide nanoparticles (IONPs), ferric carboxymaltose (FeC), or phosphate buffer saline (PBS, as a control). **b,** Hepcidin-ferroportin axis in livers of lactating mice with administration of IONPs, FeC, or PBS.

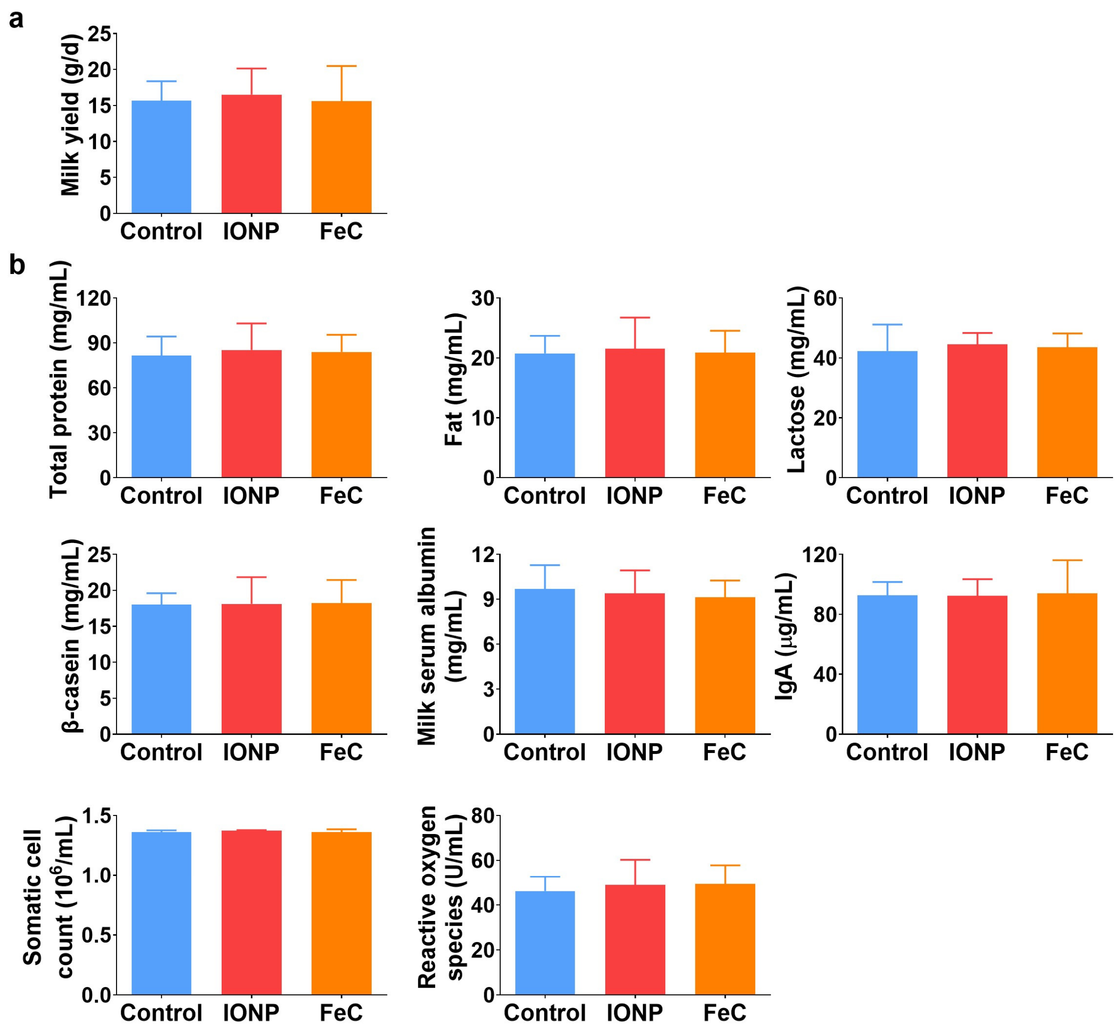

Figure S3. Milk yield and milk composition of lactating mice with/without iron supply. (a) The milk yield of mice with administration of iron oxide nanoparticles (IONPs), ferric carboxymaltose (FeC), or phosphate buffer saline (PBS, as a control) was measured during the peak milk production period (lactating days 10 and 11). (b) The milk composition (total protein, fat, lactose, β-casein, milk serum albumin, IgA, somatic cell count, and reactive oxygen species) was measured during the peak lactation stage. The data are presented as the mean ± standard deviation (n = 6).

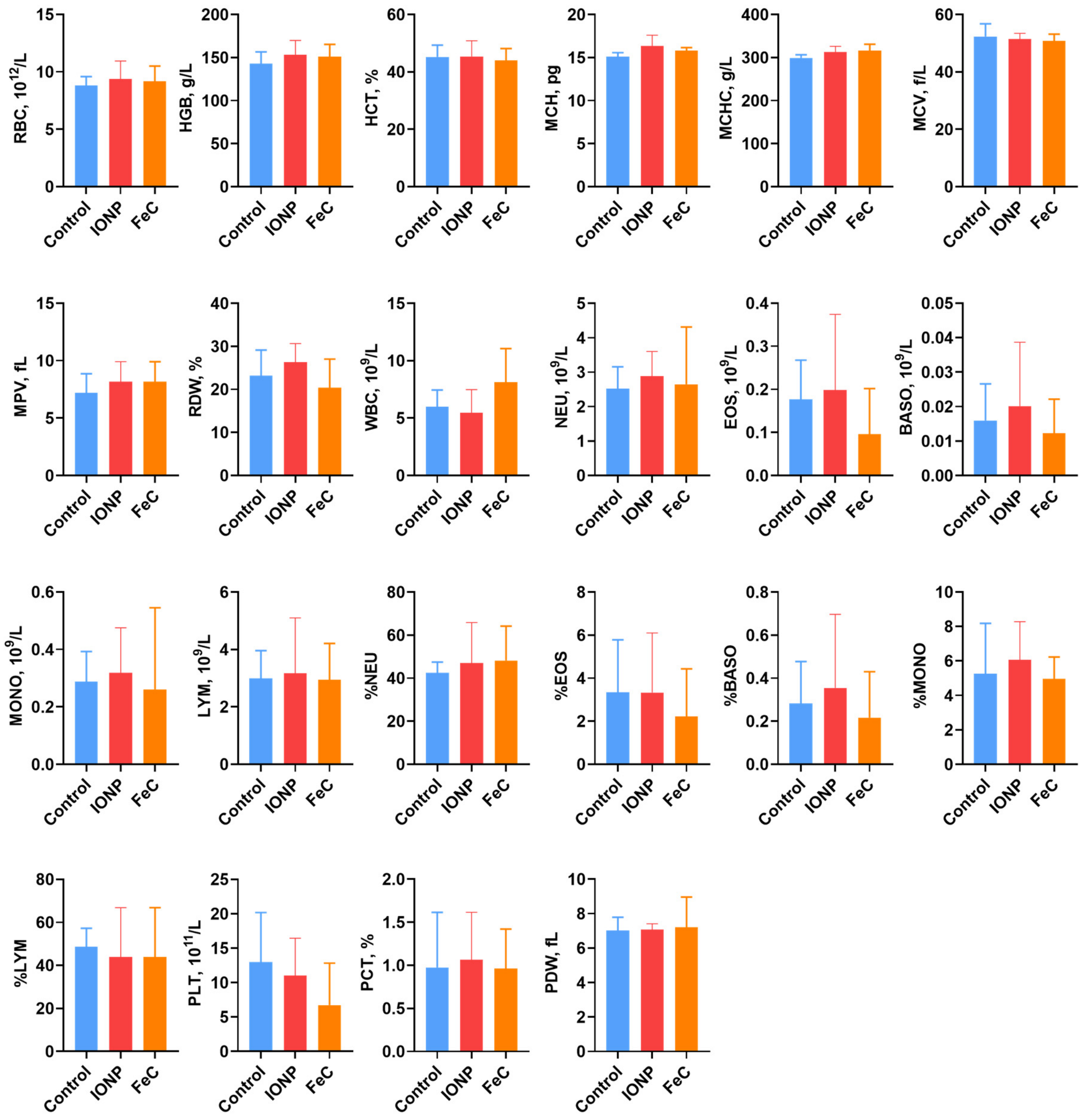

Figure S4. Hematology analysis of lactating mice with/without iron supply. Hematological indices of lactating mice treated with iron oxide nanoparticles (IONPs), ferric carboxymaltose (FeC), or phosphate buffer saline (PBS, as a control) were measured on experiment day 11. Data are presented as mean ± standard deviation (n = 6).

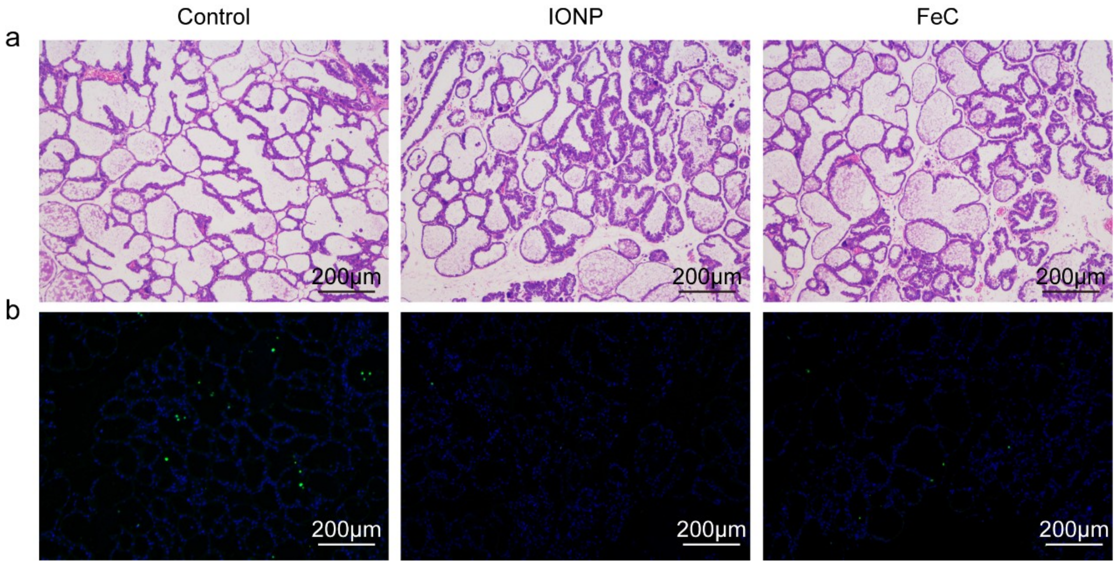

Figure S5. Pathological and apoptosis analysis of mammary gland with/without iron supply. a, Pathological analysis of mammary glands with/without iron supply by Hematoxylin-eosin staining. **b**, Apoptosis analysis of mammary glands with/without iron supply by immunofluorescence TUNEL staining.

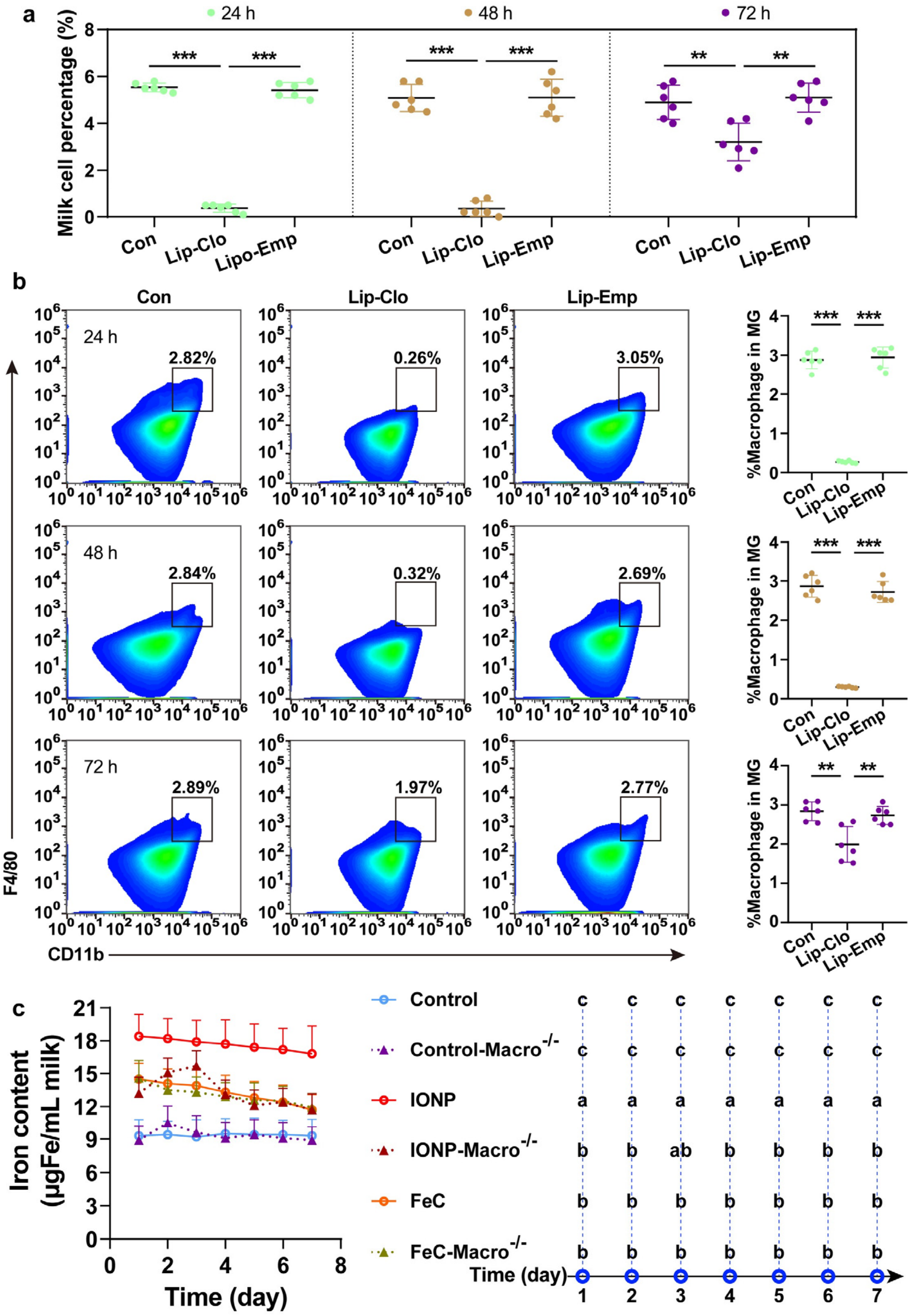

Figure S6. Milk iron levels responding to iron supplement in a macrophage depletion mouse model. Macrophage depletion was performed on lactating mice (lactation day 1) by intraperitoneally injected with clodronate liposomes (Lip-Clo), empty liposomes (Lip-Emp), or phosphate buffer saline (control group, Con). The blood and mammary gland were collected 24, 48, and 72 h after injection for the measurement of macrophage. **a**, The changes in the proportions of monocytes (form of macrophages in blood) in the blood were examined by a hematology autoanalyzer. **, and *** denote statistical significances, $P < 0.01$, $P < 0.001$, respectively. **b**, The changes in the proportions of macrophages in the mammary gland were examined by flow cytometry. Left: typical macrophage profiles; right: detailed cell numbers. **, and *** denote statistical significances, $P < 0.01$, $P < 0.001$, respectively. **c**, Milk iron content influenced by intravenous iron oxide nanoparticles (IONPs, 2.8 mg Fe·kg^{-1}), ferric

carboxymaltose (FeC, 2.8 mg Fe·kg^{-1}), or phosphate buffer saline (PBS, as a control) injection as a function of injection days (corresponding to lactation day 2-8) in macrophage-depletion (Macro$^{-/-}$) or wild-type mice. Left: milk iron contents; right: significant differences (same letters mean no significant difference, different letters ("a", "b", "c") mean significant difference). The data are presented as the mean ± standard deviation (n = 6).

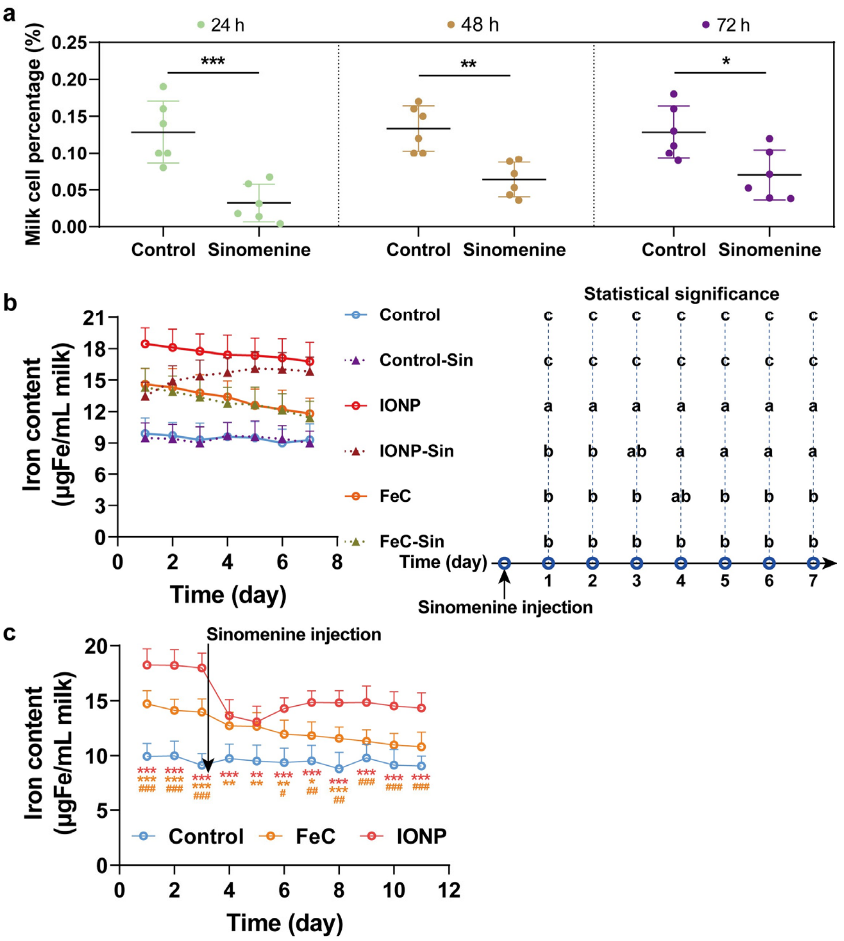

Figure S7. Milk iron levels responding to iron supplement in macrophage migration inhibition mouse models. a, The changes in the proportions of macrophages in the milk, which are migrated from mammary-resident macrophages. Macrophage migration inhibition was performed on lactating mice (lactation day 1) by intraperitoneally injected with sinomenine hydrochloride. The milk was collected 24, 48, and 72 h after injection for the measurement of milk macrophage by flow cytometry. Gating strategy seen in Figure S12. *, **, and *** denote statistical significances, $P < 0.05$, $P < 0.01$, and $P < 0.001$, respectively. **b**, Milk iron content influenced by intravenous iron oxide nanoparticles (IONPs, 2.8 mg $Fe \cdot kg^{-1}$), ferric carboxymaltose (FeC, 2.8 mg $Fe \cdot kg^{-1}$), or phosphate buffer saline (PBS, as a control) injection as a function of sinomenine hydrochloride injection days (lactation day 2-8) in macrophage-inhibition (Sin) or wild-type mice. Here, macrophage migration inhibition was induced 24 h before the IONP, FeC, or PBS administration (lactation day 1). Left: milk iron contents; right: significant differences (same letters mean no significant difference, different letters ("a", "b", "c") mean significant difference). **c**, Milk iron content influenced by IONPs (2.8 mg $Fe \cdot kg^{-1}$), FeC (2.8 mg $Fe \cdot kg^{-1}$), or phosphate buffer saline (PBS, as a control) injection as a function of lactation days (1-11). Macrophage migration inhibition was performed 72 h after the IONP, FeC, or PBS administration as indicated in the figure. In comparison of IONP vs Control (red color) or FeC vs Control (orange color): *, **, and *** denote statistical significances, $P < 0.05$, $P < 0.01$, and $P < 0.001$, respectively; in comparison of IONP vs FeC (orange color): #, ##, and ### denote statistical significances, $P < 0.05$, $P < 0.01$, and $P < 0.001$, respectively. The data are presented as the mean ± standard deviation (n = 6).

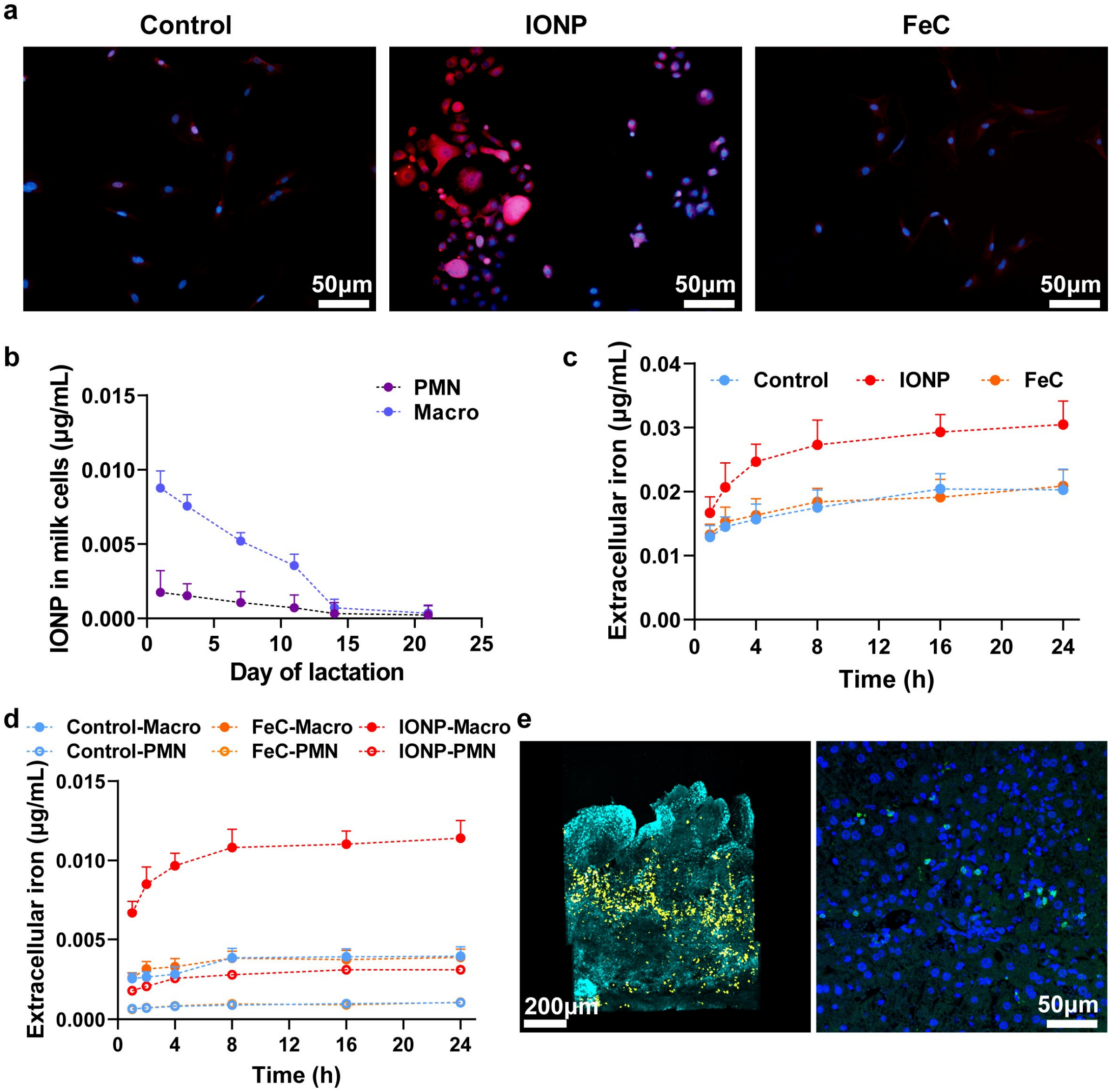

Figure S8. Intracellular iron and secreted iron of milk cells from lactating mice with/without iron supply. **a**, Intracellular iron of milk cells from lactating mice treated with iron oxide nanoparticles (IONPs), ferric carboxymaltose (FeC), or phosphate buffer saline (PBS, as a control) was measured by Ferro-orange staining. **b**, IONP content in main mammary iron carriers including milk macrophages (Macro) and neutrophils (PMN). **c**, Secreted iron of milk cells from lactating mice treated with IONPs, FeC, or PBS was measured by inductively coupled plasma-mass spectrometry. **d**, Secreted iron of milk Macro and PMN from lactating mice treated with IONPs, FeC, or PBS was measured by inductively coupled plasma-mass spectrometry. **e**, The cross-foster maternal GFP+ cells are labelled in the small intestine and liver of the pups.

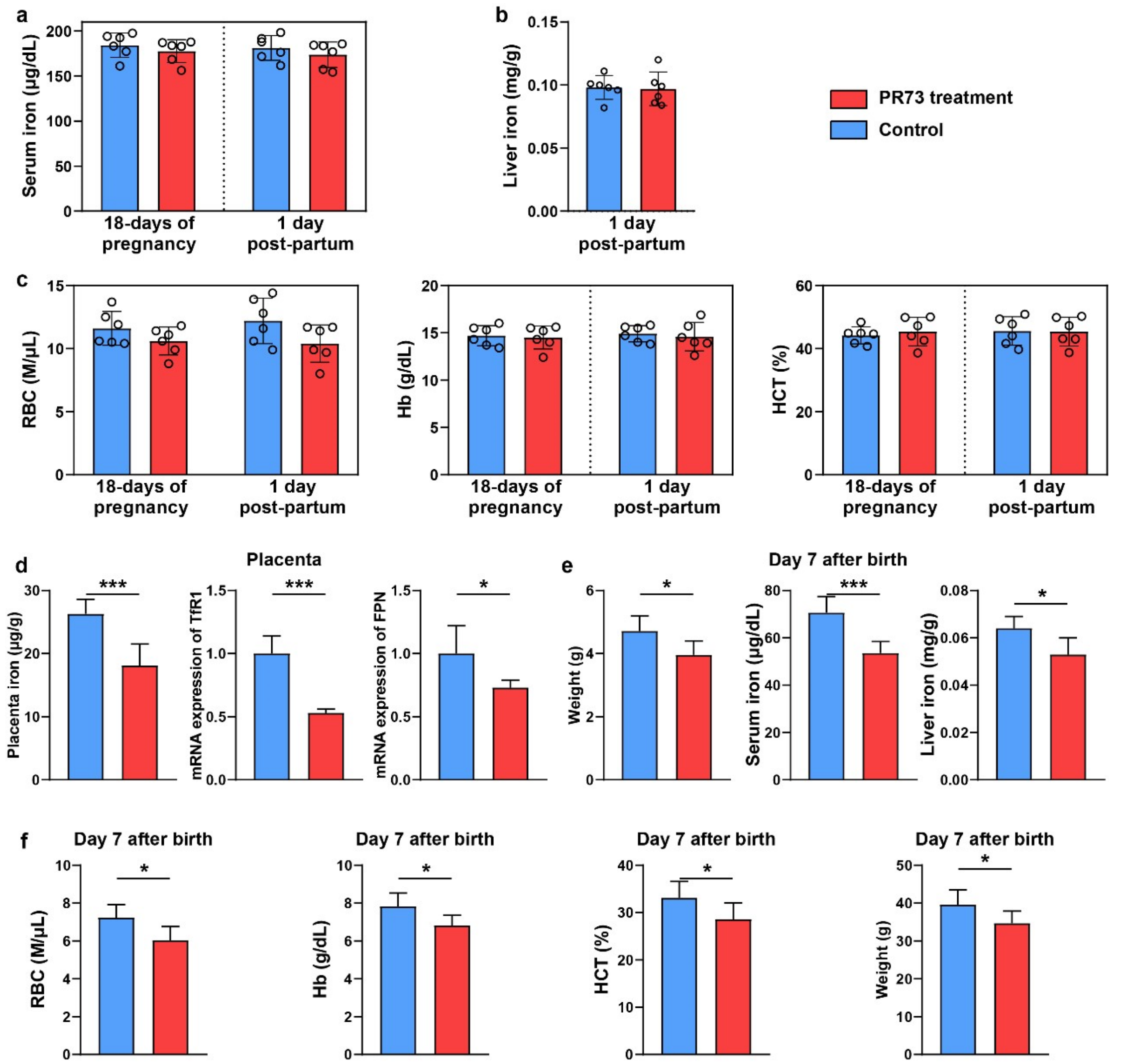

Figure S9. Effects of maternal minihepcidin-mediated iron status, anemia outcome, and hematological parameters in both mothers and offspring. **a**, Serum iron of minihepcidin (PR73-treated) and control (PBS-treated) mice at day 18 of pregnancy and at 1 d postpartum. **b**, Liver iron of minihepcidin and control mice at 1 day postpartum. **c**, Red blood cells (RBC), hemoglobin (Hb), and hematocrit (HCT) levels of minihepcidin and control mice at day 18 of pregnancy and at 1 d postpartum. **d**, Effects of hepcidin-mediated maternal iron restriction on the placenta, including placenta iron, mRNA expression of *TfR1* and *FPN* on embryonic day 18.5. **e**, Effects of hepcidin-mediated maternal iron restriction on neonatal anemia outcome, including weight, serum iron, liver iron and **f**, hematological parameters on day 7 after birth. Statistics used: unpaired *t* test. *, **, and *** denote statistical significances, $P < 0.05$, $P < 0.01$, and $P < 0.001$, respectively.

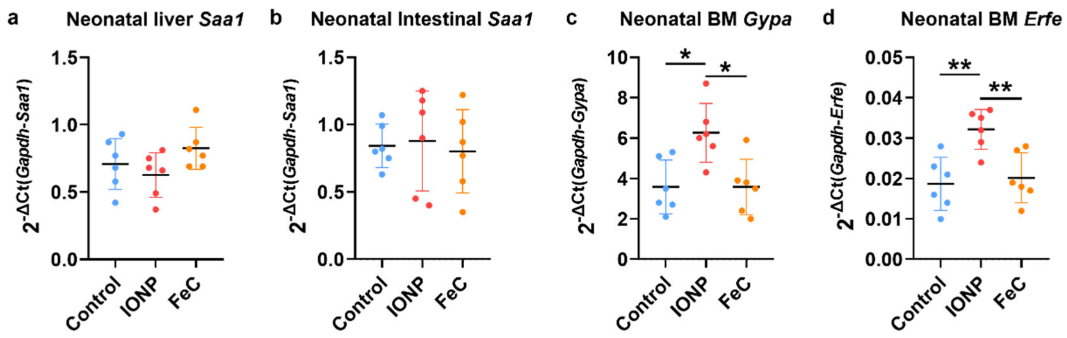

Figure S10. Anemia neonatal response to iron supply. a, Anemia neonatal liver *Saa1* mRNA expression. **b**, Anemia neonatal intestinal *Saa1* mRNA expression. **c**, Anemia neonatal bone marrow (BW) mRNA expression of the erythroid surface marker glycophorin A (*Gypa*). **d**, Anemia neonatal BW mRNA expression of erythroferrone (*Erfe*). Statistics used: one-way ANOVA. *, and ** denote statistical significances, $P < 0.05$, and $P < 0.01$, respectively.

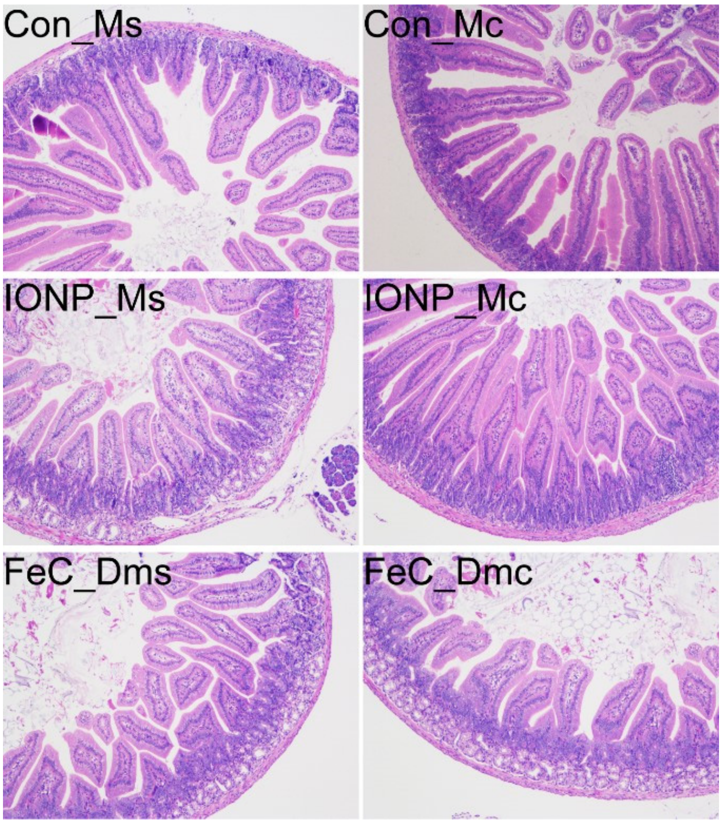

Figure S11. Morphology of neonatal mice with late-onset sepsis modeled by gut-residing *Escherichia coli* and treatments of directly oral FeC mimicking the equivalent iron supplied by milk cells (FeC_Dmc, 10.3 mg Fe·mL^{-1}) and milk supernatant (FeC_Dms, 5.8 mg Fe·mL^{-1}) from iron oxide nanoparticles (IONPs) treated mothers, and cells or supernatant from milk of lactating mice treated with IONPs, or phosphate buffer saline (PBS, as a control).

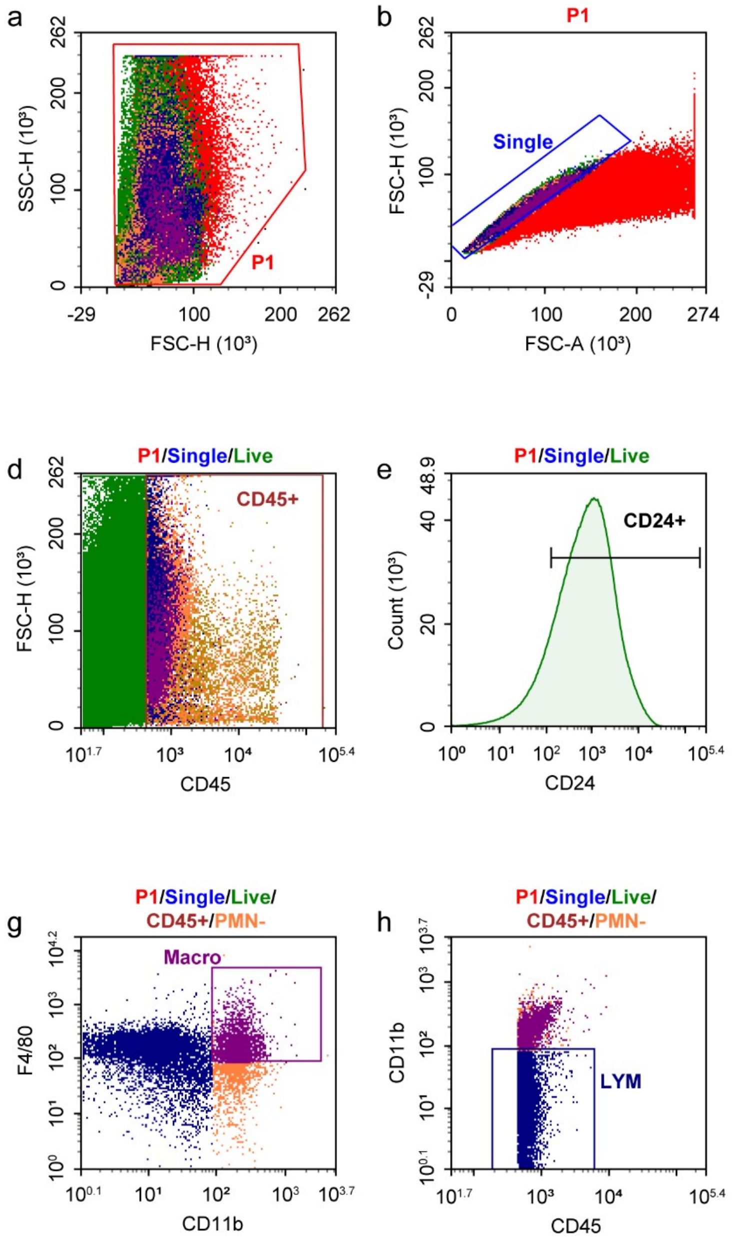

Figure S12. Gating strategy for flow cytometry analysis of cell populations in milk. a, Cells in milk were confirmed by forward scatter (FSC) and side scatter (SSC) analysis. **b,** Single cells were separated by FSC-A and FSC-H. **c,** Quantification of live cells was performed by exclusion of dead/dying cells by the 4',6-diamidino-2-phenylindole (DAPI). **d,** Single distributed $CD45^-$ and $CD45^+$ cells were separated from live cells using CD45 marker. **e,** The mammary epithelial cells were separated from single distributed $CD45^-$ cells and confirmed with CD24 high expression. **f,** Neutrophils (PMN) were separated from $CD45^+$ cells using Ly6G markers. **g,** The macrophages (Macro) were separated from non-PMN cells using CD11b and F4/80 markers. **h,** The lymphocytes were separated from non-PMN cells using CD45 and CD11b markers.

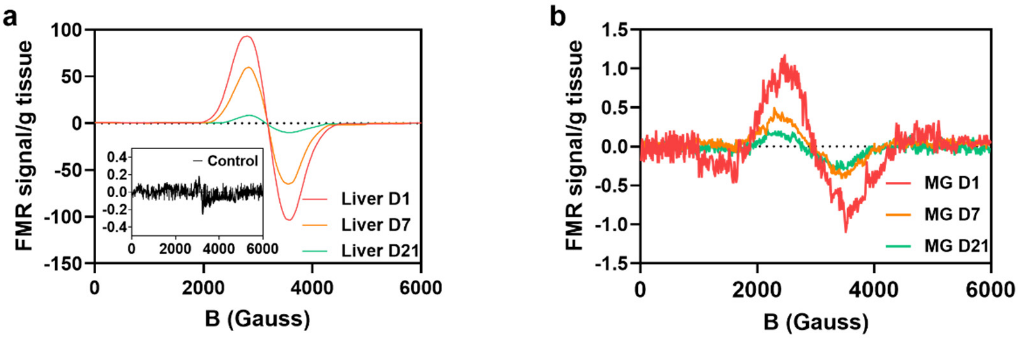

Figure S13. Representative ferromagnetic resonance (FMR) spectra of iron oxide nanoparticles (IONPs) in dried liver samples (a) and mammary samples (MG, b) on days (D) 1, 7, 21 after intravenous IONP injection to lactating mice. The intravenous IONP treatment was performed on day 1 in milk.
